# Characterization of occupational exposure to airborne particles and bioaerosols in dental clinics

**DOI:** 10.1093/annweh/wxaf073

**Published:** 2025-11-04

**Authors:** Rubiyat E Islam, Lina Wik, Vibeke E Ansteinsson, Pål Graff, Shan Zienolddiny-Narui, Torunn K Ervik

**Affiliations:** National Institute of Occupational Health (STAMI), Gydas vei 8, 0363 Oslo, Norway; Department of Public Health and Interdisciplinary Health Sciences, Institute of Health and Society, Faculty of Medicine, University of Oslo, Forskningsveien 3A, 0373 Oslo, Norway; National Institute of Occupational Health (STAMI), Gydas vei 8, 0363 Oslo, Norway; Department of Public Health and Interdisciplinary Health Sciences, Institute of Health and Society, Faculty of Medicine, University of Oslo, Forskningsveien 3A, 0373 Oslo, Norway; Oral Health Centre of Expertise in Eastern Norway (OHCE), Sørkedalsveien 10A, 0369 Oslo, Norway; National Institute of Occupational Health (STAMI), Gydas vei 8, 0363 Oslo, Norway; National Institute of Occupational Health (STAMI), Gydas vei 8, 0363 Oslo, Norway; National Institute of Occupational Health (STAMI), Gydas vei 8, 0363 Oslo, Norway

**Keywords:** aerosol, bacteria, dental environment, dental treatment, fungi, microorganisms, respirable particles, work exposure

## Abstract

Occupational exposure to airborne particles and bioaerosols in dental clinics is a potential hazard to dental health workers. Current studies on airborne particles and bioaerosols in dental clinics are limited and methodologically diverse, leaving gaps in the understanding of airborne particles in real-life dental settings. The aim of the study was to investigate the size, concentration, and composition of particles produced during dental procedures, and determine the exposure levels of dental personnel to respirable particles and bioaerosols in dental clinical environments with different characteristics. The study included two conventional dentist offices and one specialty clinic. The number concentration and size distribution of particles released during different dental procedures were monitored in real-time in dental procedure rooms. Personal samplers were used in parallel to collect the respirable and inhalable particle fractions. Total bacterial and total fungal DNA concentrations were quantified in the inhalable particle fraction by droplet digital polymerase chain reaction. Particle morphology and chemical composition were analyzed using scanning electron microscopy. The highest geometric mean value of the respirable particle mass concentration (0.06 mg/m^3^) was below the Norwegian occupational exposure limit for respirable dust of 5 mg/m^3^. Real-time sampling indicated that particle number concentrations were elevated during working hours in two clinics, with peak levels observed in one clinic coinciding with air polishing activities. The results also showed significant variations in bacterial and fungal DNA concentration levels (*P* < 0.0001). Many collected particles originated from powders used in dental treatments. Despite low respirable particle mass concentrations, increased levels of ultrafine particles during dental procedures highlight potential health risks to dental professionals. These findings also underscore the importance of advanced ventilation and safety measures to mitigate occupational exposure in dental environments.

What’s Important About This Paper? This study observed differences in exposures to particles and bacterial and fungal DNA among dental clinics, with some clinics showing variability in exposures throughout the day and among procedures and others not. Scanning electron microscopy demonstrated exposure to tooth polishing powders. While respirable particle mass concentrations were below occupational exposure limits, the increased number concentration of ultrafine particles, presence of microbes and polishing materials warrant further study of occupational exposures and risks among dental care personnel.

## Introduction

Dental care involves a series of procedures characterized by the use of diverse and chemically complex dental materials in combination with rotatory instruments. Splatters and aerosols are typically generated during such procedures using dental turbine handpieces, air-water syringes, and ultrasonic scalers (Veena et al. 2015). These aerosols can be inhaled and deposit in the respiratory tract. Studies from several countries show examples of dental health workers who reported respiratory and systemic diseases, which may have been linked to exposure during clinical work (Kerosuo et al. 2000; Stoeva 2021). Similarly, dentists may have an increased incidence of idiopathic pulmonary fibrosis, possibly caused by exposure in the occupational setting (Nett et al. 2018; Fechter-Leggett et al. 2025). Additionally, dental staff may experience higher incidence of certain airway infections than the general population (Hamilton et al. 2021; Shields et al. 2021; Mikhail et al. 2023). Exposure of dental health professionals (dentists, dental hygienists, dental health secretaries) to dental dust and pathogenic microorganisms is therefore of concern.

Increased concentrations of micrometer-sized, sub-micron (<1 µm) and nanoparticles (<0.1 µm) have been found in dental clinics in several studies, and the generation of these particles was linked to dental procedures including drilling and grinding of ceramics, metals and composite materials (Sotiriou et al. 2008; Van Landuyt et al. 2012, 2014; Polednik 2014; Chatoutsidou et al. 2021; Połednik 2021). Nanoparticles are used extensively as fillers in dental composite materials to improve material properties (Van Landuyt et al. 2012; Bogdan et al. 2014; Van Landuyt et al. 2014; Bradna et al. 2017; Schmalz et al. 2018). Furthermore, prophylactic treatments using powders such as sandblasting and polishing may also contribute to particle generation.

Bioaerosols are airborne materials from living sources, containing both viable and non-viable constituents (Ghosh et al. 2015). Aerosolized microorganisms originating from oral sources, such as dental plaque, calculus, blood, and saliva, mixed with air-water mist formed by rotatory instruments spread as bioaerosols in dental operatory areas (Hallier et al. 2010; Schmalz et al. 2018). This can lead to cross-infection among staff and patients (Szymańska 2007). In addition, the water supply, ventilation system, and other indoor environmental factors may influence the composition, distribution, and levels of bioaerosols in dental clinics (Leggat and Kedjarune 2001).

Studies on particle and bioaerosol concentration levels in dental clinics have to date been conducted with methodological heterogeneity. Previous measurements in dental clinics have mostly been performed using direct reading instruments and particle characterization is often lacking (Helmis et al. 2007; Van Landuyt et al. 2012, 2014; Polednik 2014; Lang et al. 2018; Chatoutsidou et al. 2021; Połednik 2021). In addition, studies have often focused on assessing particle generation, size, and dispersion in controlled environments rather than in routine clinical settings (Bogdan et al. 2014; Bradna et al. 2017; Nulty et al. 2020; Shahdad et al. 2021; He et al. 2022; Kumar et al. 2023). Moreover, despite evidence indicating that certain dental procedures generate high concentrations of particles and bioaerosols, the actual personal exposure level of dental staff is not well-studied. To address these gaps, this study aimed to use mass-based personal sampling equipment to evaluate the individual exposure level to the respirable particle fraction in dental clinics together with bioaerosol measurements, which in combination with direct reading instruments gives information on the exposure of dental health professionals during routine clinical procedures. In addition, the aim was to thoroughly characterize particle types and chemical composition in the respirable fraction collected on the filters by scanning electron microscopy.

## Materials and methods

### Sampling sites

Air sampling measurements were conducted at three dental clinics, hereafter referred to as Dental Clinic 1, 2, and 3, respectively (Table 1, Table S1, Fig. S1). All three clinics were in the Oslo and Asker municipalities, Norway. A snowball sampling technique was used to identify and include the study sites. Dental Clinic 1 was located in a modern building with a mechanical ventilation system. Dental Clinic 2 was situated in an older building with a mechanical ventilation system. In contrast, Dental Clinic 3 was in an old apartment building relying on natural ventilation by the opening of windows. The first sampling took place from August to September 2023 (3 consecutive days/week) at Dental Clinic 1, and the second and third sampling from in March and May 2024, two weeks in Dental Clinic 2 and Dental Clinic 3 (4 days/week in each clinic), respectively. This study involved personal air sampling and no biological data or health data from human subjects were collected. Participation in the study was voluntary. Patients were informed about sampling campaigns, and the equipment could at any time be removed in case of patient-perceived anxiety or stress. Based on the evaluation (application no. 940003) from the Regional Committee for Medical and Health Research Ethics in South-Eastern Norway (REK sør-øst), the project falls outside the scope of the Health Research Act, and no ethical approval from REK was required for its implementation.

**Table 1.** Characteristics of the sampling campaign.

Dental clinic	1	2	3
Number of campaign days	9	4	4
Total dental professionals included	9	5	3
Dentists	4	3	2
Dental health secretaries	4	2	1
Dental hygienist	1	0	0
Total respirable particle samples	18	16	9
Total bioaerosol samples	18	16	9
Stationary samplers			
SMPS	yes	yes	yes
APS	yes	yes	yes
Ventilation	mechanical	mechanical	natural
Average patients/treatment room/day	2	8	7
Total procedures^a^/treatment room	38	131	78
Prophylactic % (*n*)	37 (14)	77 (101)	67 (52)
Repair and restoration % (*n*)	34 (13)	21 (28)	27 (21)
Surgical and tooth extraction % (*n*)	29 (11)	1 (1)	0 (0)
Orthodontic % (*n*)	0 (0)	1 (1)	6 (5)

Procedural grouping: prophylactic (examination, pumice powder polishing, sandblasting, ultrasonic scaling, and air polishing); Repair and restoration (composite filling restoration, crown restoration, root canal treatment); Surgical (implant, surgical tooth extraction, and nonsurgical tooth extraction); Orthodontic (work with aligners, retainers and bite splints).

SMPS, scanning mobility particle sizer; APS, aerodynamic particle sizer.

^a^Total number of procedures during the campaign

### Air sampling methods

#### Stationary instruments

Direct reading stationary instruments were used to measure airborne particle size distribution and number concentration continuously during working days. The Scanning Mobility Particle Sizer (SMPS, model 3938, TSI Incorporated, Minnesota, USA) was measuring particles ranging from mobility diameter of (*d*_mob_) 16.8 to 593 nm. An Aerodynamic Particle Sizer (APS, model 3321, TSI Incorporated, Minnesota, USA) measured particle size distribution and number concentration within the aerodynamic diameter range of 0.542 to 19.8 µm (*d*_ae_). The stationary instruments were positioned close to (approximately 1 to 1.5 m) the dental operating chairs of the respective treatment rooms (Fig. S1). In addition, background particle measurements were recorded with these same instruments during weekends. Different dental treatment procedures (Table S2) and the duration of the treatment procedures were recorded to evaluate the treatment procedure-specific occupational exposure and coordinate with the direct vicinity data from stationary instruments in the dental clinics.

#### Personal samplers

At the beginning of each working day, participating workers in the dental clinics were provided with prepacked backpacks containing sampling equipment (Fig. S2). Each participant carried (i) a respirable particle cyclone (JS Holdings, Sevenage, UK) equipped with 37 mm polyvinyl chloride filters with pore size of 5.0 µm (Merck Millipore KGaA, Darmstadt, Germany) and (ii) a conical inhalable sampler (CIS) (JS Holdings, Hertfordshire UK) containing 37 mm polycarbonate (PC) membrane filters with 0.8 µm pore size (Merck Millipore KGaA, Darmstadt, Germany). Air pumps (Casella Apex2, Bedford, United Kingdom) operating at an average airflow of 2.2 and 3.5 L/min for the respirable and inhalable sampler, respectively were carried inside the backpack, and the sampling inlets placed within the participant's breathing zone. The airflow of the sampling devices was adjusted before and after sampling with a rotameter (TeknolabAS, Akershus, Norway). The sampling time covered the working shift period with an average sampling time of 360 ± 120 min per shift.

The personal sampling devices were retrieved from the study participants at the end of the working shift. Following collection, exposed air samplers and corresponding filters were transported in individual sealable plastic bags. CIS cassettes were handled in a Laminar Air Flow (LAF) bench and cleaned with detergent and 70% ethanol before and between samplings. Cassettes were transported in individual zip-lock bags, and exposed filters were dried inside a LAF bench and stored in sterile 15 mL tubes at 4 °C until DNA extraction and analysis. For each clinic, two CIS cassettes were prepared for field blanks and kept sealed during transportation and sampling.

#### Gravimetric analysis

For gravimetric analysis of the respirable fraction, filters were equilibrated in a climate-controlled room for at least 48 h at a mean temperature of 20 ± 1 °C and relative humidity of 39 °C ± 2. The gravimetric analysis for particles per filter was done using a microbalance (Sartorius AG, MC21, Göttingen, Germany). Blank filters were prepared for every 10th field sample. The limit of detection (LOD) was calculated to be threefold the standard deviation of blank filters at 0.03 mg/filter.

#### Bioaerosol filter elution and DNA extraction

The DNA extraction method used in this study was based on the protocols described by Straumfors et al. (2019). Briefly, the filters were washed with 0.1% PBST made of 10 mL of phosphate-buffered saline (Thermo Fisher Scientific Inc., Massachusetts, USA) and 0.1% Tween 20 (Sigma Aldrich, Missouri, USA). The samples were then sonicated for 3 min, mildly vortexed for 10 s, and centrifuged at 2,500 × *g*  for 30 s. Filters were removed, and filter eluates were re-centrifuged at 3,500 g at 4 °C for 30 min. The supernatant was transferred to 1.5 mL tubes. Samples were lysed using liquid nitrogen (N_2_) and heat (90 °C) treatment, followed by bead beating (Mini Beadbeater-96; BioSpec Products Inc.). Furthermore, the samples were incubated before (at 300 rpm at 65 °C for 15 min) and after (at 300 rpm at 65 °C for 45 min) the addition of 254 µL cetyltrimethylammonium bromide buffer (PanReac AppliChem, Darmstadt, Germany) and RNase A, and DNA was isolated using the DNeasy mini-spin column separation kit (DNeasy plant kit, Quiagen GmbH, Hilden, Germany), following the manufacturer's instructions. DNA was also isolated from filters used as field blanks. DNA concentration was evaluated by a Qbit 4 fluorometer (Thermo Scientific DE, USA) and the DNA samples were stored at −20 °C. Template DNA concentrations were low; hence samples were not further diluted.

#### Detection of bacterial and fungal DNA

Due to low template DNA concentrations, droplet digital polymerase chain reaction (ddPCR) was chosen to directly amplify and quantify the bacterial and fungal DNA to assess the personal exposure to these types of bioaerosol. The PCR reaction mixture (16 µL) was prepared from 10 µL EvaGreen Super Mix, 0.2 µL (25 µM) of each forward and reverse primer pair respectively for bacteria: P11P/P13P (Widjojoatmodjo et al. 1995) and fungi: FF390/FR1 (Chemidlin Prévost-Bouré et al. 2011), 5.6 µL PCR grade water, and 4 µL genomic template DNA. The reaction mixture volume was partitioned into droplets before running PCR using a Bio-Rad QX200 droplet generator (Bio-Rad Laboratories Inc. CA, USA). During amplification, the bacterial DNA was denatured at 95 °C for 5 min, followed by 40 amplification cycles, alternating between 95 °C for 30 s at the first step, 1 min at 60 °C, and 1 min and 30 s at 70 °C at the later step. A two-step stabilizing step was implemented for 5 min at 4 °C and 5 min at 90 °C. Fungal DNA was denatured at 95 °C for 5 min, amplified for 40 cycles rotating among 30 s at 95 °C, 30 s at 50 °C, and 1 min at 60 °C, and stabilized in two steps at 4 °C for 5 min and 90 °C for 5 min. Quality scores of PCR materials were evaluated with Bio-Rad QX200 droplet software (Bio-Rad Laboratories Inc. CA, USA). The ddPCR threshold for identifying positive droplets was set at 20,000 amplitudes for bacteria and 12,000 amplitudes for fungi to uphold the high quality of positive droplets. This threshold value was used as a standard for determining the presence of specific target organisms in the samples.

#### Scanning electron microscopy

Scanning electron microscopy (SEM) was used to investigate the size and chemical composition of individual respirable particles collected in the three dental clinics. Initially, the three PVC filters containing the highest respirable particle mass collected from each dental clinic were selected. The filters were washed with ethanol mixed with distilled water solution and sonicated for 4 min. Then the particle solution was filtrated using a sinter in a Büchner funnel through PC filters with a pore size of 0.8 µm. Approximately 8 × 8 mm sized pieces from every filter were cut out and mounted on 10 mm aluminum stubs covered with double-sided carbon adhesive discs. Carbon cement (Leit-C, Agar Scientific Ltd.) spots were added to the corner of the stub to ensure optimal conductivity between the filter and stub. Thereafter, the specimens were coated with platinum (Pt) with a thickness of 10 nm in a Cressington 208 h sputter coater (Cressington Scientific Instruments Ltd., Watford, United Kingdom) to provide a conductive layer for imaging in SEM. The particles were imaged by SEM using a Hitachi SU6600 field emission instrument (Hitachi, Tokyo, Japan) equipped with a Bruker energy dispersive X-ray (EDX) detector. The working distance was set at 10 mm and an acceleration voltage of 15 keV was used. On each filter, 57 to 102 particles were analyzed by EDX by point analysis. For comparison, material samples from the products used in the dental clinics were also investigated in SEM. The following powders were obtained: Pumice stone powder (Produits Dentaires AS, Vevey, Switzerland), a sodium bicarbonate (NaHCO_3_)-based air polishing (air flow) powder (EMS, Nyon, Switzerland) and an aluminum oxide (Al_2_O_3_) for sandblasting procedures (Rønvig Dental Mfg. AS, Daugaard, Denmark).

### Statistical analysis

Data analysis and visualization was done using GraphPad Prism version 10.2.3 for Windows (GraphPad Software, California USA, www.graphpad.com) or for linear mixed models (LMMs) RStudio v4.2.2 (R core team, 2022) along with the lme4 package (v1.1-31; Bates et al. 2015). Descriptive analysis was performed on respirable particle mass (mg/m^3^) and total bacterial and total fungal DNA concentrations (copies/m^3^), which were shift-duration time-weighted average results. The distribution of the response variables was assessed using QQ-plots and skewness, and the data was log-transformed prior to statistical analysis to enhance normality. Samples with particle mass concentrations under the LOD were substituted by LOD/2 (Ogden 2010). Comparison of respirable particle and DNA concentrations among the three dental clinics were assessed with log-transformed data by ANOVA followed by Tukey's multiple comparison test. The Pearson correlation coefficient was used to evaluate the correlation between particle mass concentration and total microbial DNA concentration (bacteria and fungi combined).

When evaluating the associations between respirable particle mass concentration, total bacterial and total fungal DNA concentrations and worker classification, LMMs were used to account for dependencies arising from repeated measurements within dental clinics. The response variables included particle concentration, fungal DNA, and bacterial DNA, with profession (dentist and dental health secretary) specified as a fixed effect and dental clinic as a random effect. The residuals from the models were further evaluated using QQ-plots to assess their normality. A significance threshold of *P* < 0.05 was established for all statistical analyses.

## Results

Seventeen dental professionals from three dental clinics participated in the study, including dentists, dental health hygienists and dental health secretaries (Table 1, Table S1). The average number of patients per day per treatment room was 2, 8, and 7 in Dental Clinics 1, 2, and 3, respectively. Treatments were subdivided into each procedure before scoring the number of performed procedures. Therefore, the total number of performed procedures is higher than the number of treated patients (Table 1). While Dental Clinic 1 offered both surgical and general dental treatment procedures, the other two dental clinics mostly focused on general dental treatment procedures. 77% of the procedures in Dental Clinic 2 were prophylactic.

### Respirable particle mass concentration

A total of 43 person-borne respirable particle samples (Table 2, Table S1) were collected. The geometric mean (GM) for respirable particle mass concentration in Dental Clinic 1 was 0.04 mg/m^3^, whereas, in Dental Clinic 2 and Dental Clinic 3, GM was 0.06 mg/m^3^ and 0.03 mg/m^3^ respectively (Table 2). Three out of 18 samples from Dental Clinic 1 had unexpectedly high negative values due to handling errors and were not included in the inferential analyses. Eleven samples from Dental Clinic 1 and four samples from Dental Clinic 2 were under the LOD. Differences in GM particle mass concentration in the three dental clinics were not statistically significant (*P* = 0.1988, ANOVA, Fig. S3). Dental Clinic 2 had a higher GM particle concentration than Dental Clinic 1 and Dental Clinic 3, however pairwise tests showed no statistically significant differences (*P* = 0.5698 and *P* = 0.1775 respectively, Fig. S3). Additionally, a minimal variance was attributable to clinic differences in the mixed-effects model with respect to particle concentration (Table S3).

**Table 2.** Descriptive analysis of personal respirable particle concentration (mg/m^3^) and total bacterial and fungal DNA concentrations (copies/m^3^) in the three dental clinics included in the study.

Sampling sites	Sample size	Geometric mean (SD)^a^	Arithmetic mean (SD)^a^	Min-Max^a^
**Respirable particle concentration**				
Dental Clinic 1	15^b^	0.04 (2.1)	0.05 (0.06)	0.02 to 0.22
Dental Clinic 2	16^b^	0.06 (2.1)	0.07 (0.03)	0.02 to 0.11
Dental Clinic 3	9	0.03 (1.8)	0.04 (0.02)	0.01.0.07
**Bacterial DNA**				
Dental Clinic 1	18	2,143 (2.8)	3,293 (3,498)	226.4 to 15,916
Dental Clinic 2	16	2,479 (1.6)	2,737 (1,287)	788.9 to 6,480
Dental Clinic 3	9	12,087 (1.7)	13,441 (5,677)	3,829 to 22,222
**Fungal DNA**				
Dental Clinic 1	18	411.8 (1.8)	477.1 (248.3)	101.1 to 1,063
Dental Clinic 2	16	783.7 (2.0)	957.3 (605.3)	246.3 to 2,284
Dental Clinic 3	9	5,991 (2.0)	7,094 (3,623)	1,407 to 11,741

Respirable particle concentrations were collected with respirable cyclones and measured by gravimetric analysis. Bacterial and Fungal DNA was collected by personal inhalable samplers and total DNA concentration assessed by ddPCR.

^a^Geometric mean, mean, and min-max values are all given in mg/m^3^ (particle concentration) and total bacterial and fungal DNA concentration (copies/m^3^).

^b^Eleven samples from Dental Clinic 1 and four samples from Dental Clinic 2 were under the limit of detection (LOD) and were substituted by LOD/2.

### Total bacterial and fungal DNA concentration

43 person-borne bioaerosol samples (Table 2, Table S1) were collected and total bacterial and total fungal DNA concentrations were measured. Bacterial and fungal DNA exposure varied among the dental clinics and were highest in Dental Clinic 3 (GM: 12,087 copies/m^3^ and 5,991 copies/m^3^ respectively) and lowest in Dental Clinic 1 (GM: 2,143 copies/m^3^, 411.8 copies/m^3^ respectively, Table 2). Moreover, statistically significant differences in GM exposure to bacterial DNA were shown among the dental clinics (*P* < 0.0001, ANOVA, Fig. S4a). Pairwise comparisons showed that the mean bacterial DNA concentration was significantly higher in Dental Clinic 3 than in Dental Clinic 1 (*P* < 0.0001) and Dental Clinic 2 (*P* < 0.0001, Fig. S4a). A similar pattern was observed for fungal DNA concentrations (Fig. S4b), though in pairwise tests Dental Clinic 2 also exhibited a significantly higher fungal DNA concentration than Dental Clinic 1 (*P* = 0.0223). In general, total bacterial DNA concentrations were significantly higher in the three dental clinics compared to the total fungal DNA concentration (*P* < 0.0001, ANOVA). Incorporating clinic as a random effect in LMMs revealed variability among clinics for bacterial concentration and fungal concentration (intercept estimate of 0.16 and 0.36, respectively, Table S3).

Microbial DNA concentration levels, however, were neither statistically significantly correlated with particle mass concentration (data not shown), nor to the procedural categories (Table 1 and data from multiple regression analyses are not shown).

### Direct Reading stationary instruments

**Fig. 1.**
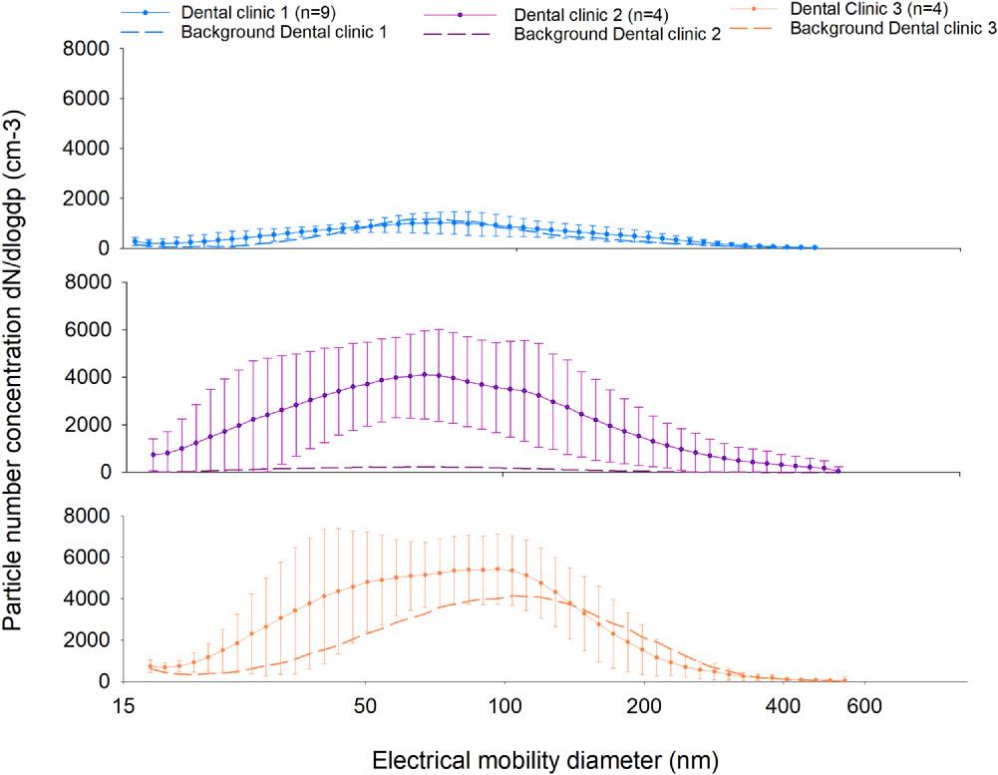
Particle size distribution represented by electrical mobility diameter in the size range of 16.8 to 593 nm (d_mob_) measured by SMPS in three dental clinics during working hours. Background levels are derived from days when the clinics were closed. Particle number concentration is shown on the *y*-axis.

The particle number concentrations measured with SMPS averaged over the working day are shown in Fig. 1. Dental Clinic 1 generally had lower number concentrations than Dental Clinic 2 and 3. Particle number concentration levels measured for Dental Clinic 1 during working hours were comparable to the background levels. For Dental Clinic 2, average working hour particle number concentrations were clearly higher than background levels for the whole size range measured, with a number concentration above 4,000/cm^3^ for ultrafine particles measuring approximately 70 nm. Background particle concentration levels in Dental Clinic 3 were elevated compared to the other clinics, and particle concentration levels during work hours were more comparable to background. However, during work hours particle number concentrations in the size range of 15 to 100 nm (nanoparticles) were elevated compared to background levels (Fig. 1).

**Fig. 2.**
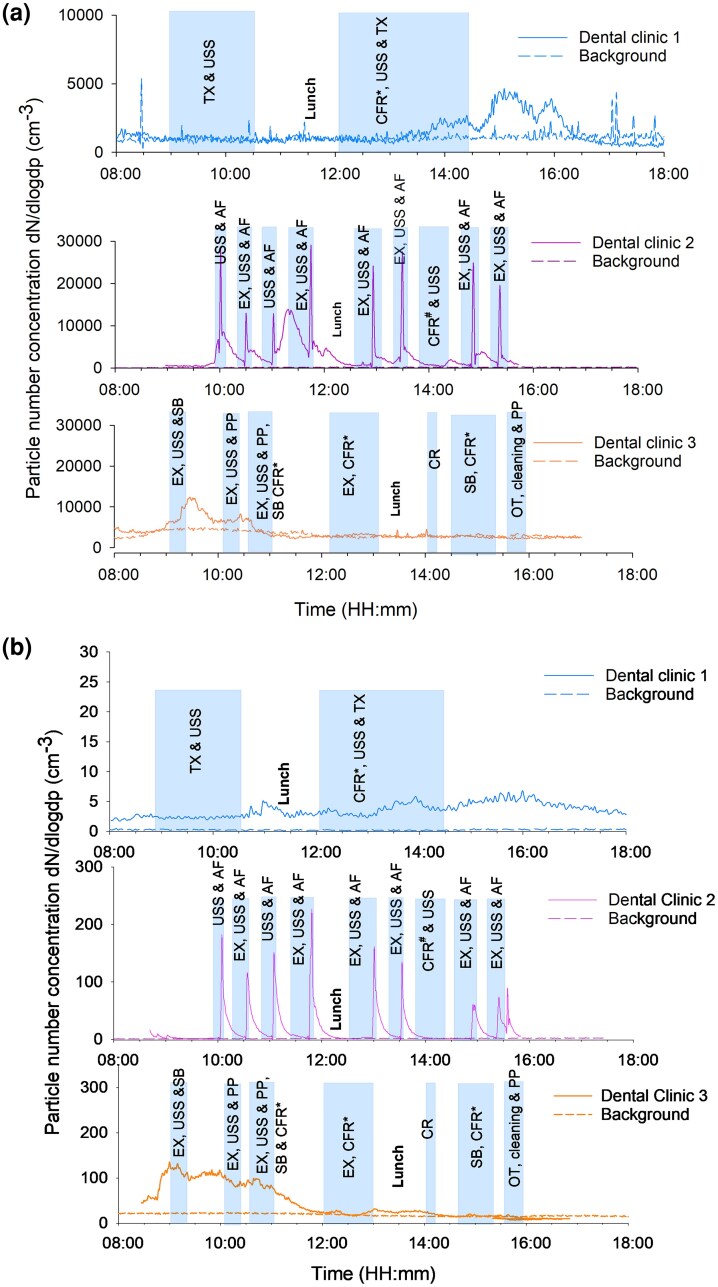
Time series of a) the total number concentration for particles in the size range of 16.8 to 593 nm (*d*_mob_) measured by SMPS, and b) number concentration of particles with size of *d*_ae_ = 1.04 µm measured with APS, during representative working days and background periods (weekends) among three dental clinics. Abbreviations of the dental treatment procedures; USS, ultrasonic scaling; AF, air polishing with air flow powder; CFR*, polishing of composite filing restoration; CFR#, drilling of composite filling restoration; CR, crown preparation; EX, examination; OT, orthodontic treatment; PP, polishing with pumice powder; SB, sandblasting with Al_2_O_3_; TX, tooth extraction.

The time-resolved graphs measured by SMPS (size range of 16.8 to 593 nm (d_mob_) and APS (here represented by a size of d_ae_ = 1.04 µm) show the impact of various dental procedures on total particle number concentrations. One representative working day is shown for the three dental clinics (Fig. 2a and b, respectively). Additional time-resolved SMPS graphs from the other days can be found in Figs. S5 to S7.

The SMPS measurements showed that certain dental treatment procedures, specifically air polishing, were associated with higher particle concentrations in Dental Clinic 2 (Fig. 2a). These procedures were performed at different timepoints during working days, resulting in particle concentrations with peaks ranging from approximately 12,000 particles/cm^3^ to 28,000 particles/cm^3^ (Fig. 2a). For Dental Clinic 3, a minor increase in the concentration of particles was noted following sandblasting treatment procedures. Dental Clinic 1 had fewer variations in dental treatment procedures and showed low particle concentrations (around 1,000 particles/cm^3^) during working periods.

The direct reading measurements from APS represented here by a size of d_ae_= 1.04 µm (Fig. 2b) showed a similar trend to the measurements with SMPS (Fig. 2a). For Dental Clinic 2, elevated concentrations of particles (50 to 240 particles/cm^3^) were generated during ultrasonic scaling and air polishing. For Dental Clinic 3, however, episodes of elevated particle concentrations were observed both during and after the treatment procedures had been performed.

### Particle characterization by SEM

Investigated particles were grouped into categories based on their morphology and on their element composition. An overview of the predominant particle types on the filters is provided in Table 3. Selected SEM images of particle types found on the collected filters are shown in Fig. S8. The pumice stone powder obtained had a ratio between aluminum (Al) and silicon (Si) of 0.3 to 0.5, potassium (K) and Si of 0.1, and 0.05 to 0.1 for sodium (Na) and Si. Dental Clinic 1 and Dental Clinic 3 used pumice powder for polishing and most inorganic particles investigated on filters from these two clinics had a composition similar to the pumice stone powder (Table 3). In Dental clinic 2 the NaHCO_3_-based powder was used as air flow powder following the ultrasonic scaling. From the safety data sheet this product also contained nanosized amorphous silica, saccharin, and flavor. In SEM, this powder was found to contain particles with the elements Na, oxygen (O) and carbon (C), in addition to Si and O containing particles. Most inorganic particles collected on the filters from Dental Clinic 2 could be related to this powder (Table 3). In the C-rich group, particles often contained only C and O, as seen in Fig. S8g. Soot was not found on the filters.

**Table 3.** Particle types found on the filters from the three dental clinics.

Elements	Particles (%)		
**Dental Clinic 1**	Filter 1 (*n* = 95)	Filter 2 (*n* = 84)	Filter 3 (*n* = 73)
Pumice stone powder particles^a^	3.2	15	49
Si-rich	1.1	14	27
Fe-rich	ND	4.8	ND
Ca-rich	3.2	2.4	4.1
C-rich	92	63	20
**Dental Clinic 2**	Filter 1 (*n* = 57)	Filter 2 (*n* = 71)	Filter 3 (*n* = 70)
Air flow powder particles^b^	46	66	58
Fe-rich	5.3	ND	1.4
Al-rich	ND	1.4	ND
Ca-rich	8.8	11	7.1
C-rich	40	21	32
**Dental Clinic 3**	Filter 1 (*n* = 89)	Filter 2 (*n* = 102)	Filter 3 (*n* = 80)
Pumice stone powder particles^a^	65	67	69
Si-rich	2.2	11	6.3
Fe-rich	2.2	2.0	1.3
Al-rich	ND	1.0	3.8
Ca-rich	2.2	2.0	ND
C-rich	28	18	20

The particles from the filters were detected and analyzed by SEM with EDX detector. The number in parentheses is the total number of particles investigated on the filter.

ND, not detected; NA, not applicable.

^a^Pumice stone powder identified based on Si, Al, Na, and K content and ratio between these.

^b^AirFlow powder identified based on the detection of Na, O, Si, and C.

## Discussion

Among three dental clinics, Dental Clinic 2 had the highest number of patients per day, and highest fraction of prophylactic treatments (77%) during the sampling campaigns. The GM of respirable particle mass concentration in Dental Clinic 2 was 0.06 mg/m^3^, which was higher than the mass particle concentration found in the other two dental clinics, although the differences were not statistically significant (Table 2; Fig. S3). Additionally, the linear mixed model (Table S3) indicated no significant contribution from the clinic to the observed variance. Notably, the large residual variance for particle concentration implies that a considerable amount of uncertainty remains in the data that is not captured by the model. Previous studies often used direct reading instruments, and the results are not directly comparable to the filter-based method used in this study. The basis of comparison is therefore scarce. In a study by Polednik, respirable concentrations ranging from 10 µg/m^3^ with episodes to above 600 µg/m^3^ were measured using stationary direct reading instruments in a dental clinic (Połednik 2021). Van Landuyt measured different PM (1 to 10 µm) sizes with a particle counter carried by dental personnel and used these values as a measure for the respirable fraction. Short time episodes with mass concentration peaks up to 10 mg/m^3^ were measured for total suspended particles during dental procedures such as polishing and contouring restorations without water (Van Landuyt et al. 2014). Previous mass-based measurements indicated mass concentrations in a range similar to the present study, using stationary equipment. The mass concentration in a dental clinic ranged from 23 to 229 µg/m^3^ and 33 to 326 µg/m^3^ for stationary measurements of PM 2.5 and PM 10, respectively (Helmis et al. 2007). The highest cumulative mass concentration measured in a dental office with a stationary cascade impactor, targeting particles in the size range of 30 nm to 10 µm, was 0.06 mg/m^3^ (Lang et al. 2018). It is important to note that a PM 10 sampler collects larger particles than a respirable sampler and a PM 2.5 sampler collects only a subfraction of the respirable fraction.

Even though the mass concentrations of respirable particles collected on filters were relatively low, the SMPS showed elevated particle number concentration in Dental Clinics 2 and 3 compared to the background concentration (during non-dental activities) for particle size range of 16.8 to 593 nm (d_mob_) (Fig. 1, lower panels). Small particles contribute less to the mass, however, will often dominate by numbers. Particle concentrations in Dental Clinic 1 were comparable to background levels (Fig. 1, upper panel). A possible explanation for this could be that the nature of the dental procedures performed during the sampling in Dental Clinic 1, which is a specialty clinic, were dominated by less aerosol-generating activities, such as tooth extraction or dental implantation (Table 1).

The time-resolved particle number concentrations measured with SMPS and APS (Fig. 2) indicated that specific dental procedures were responsible for releasing particles, especially in Dental Clinic 2. In previous studies grinding and drilling procedures were found to be the primary cause of sub-micron airborne particle release in dental offices (Sotiriou et al. 2008; Połednik 2021), whereas ultrasonic scaling and air-abrasion polishing was found to cause concentration increase of relatively large particles (>1 µm) (Połednik 2021). Grinding, drilling and polishing of composite filling restorations also took place during the sampling campaigns in this study, however, the concentration peaks shown in Fig. 2 as well as in Figs. S6 and S7 could not be linked to these processes. The reason for this could be that for most of the time water was used. Air polishing, which uses an air flow powder, was widely used in Dental Clinic 2 after ultrasonic scaling. In the present study, ultrasonic scaling was performed in all three clinics whereas the air polishing procedure was mainly performed in Clinic 2. Air polishing appears to be the main cause of the concentration peaks seen in Dental Clinic 2. It is also important to keep in mind that liquid particles formed during some of these procedures may contribute to the number counts, both for APS and SMPS.

Moreover, the ventilation system seemed to have a significant effect on particle number concentration levels in the dental clinics studied. In Dental Clinic 2, each aerosol-generating procedure resulted in a sharp increase in ultrafine (<0.1 µm) particles as well as in larger particles, here illustrated by data from measurements at 1.04 µm (d_ae_) (Fig. 2a and b, middle panels, respectively). However, as particle number concentrations returned to baseline within around 30 min, these particles were efficiently removed by the ventilation system. In contrast, in Dental Clinic 3, which used natural ventilation, particle number concentrations remained elevated for longer periods during and after the end of aerosol-generating procedures such as sandblasting (Fig. 2, lower panels, 09:00 to 11:00 hours). Thus, particles produced in the breathing zone of dental professionals and patients during procedures likely took longer to dissipate. A study investigating fallow time in mechanically ventilated environments and non-mechanically ventilated environments showed very low baseline levels and a rapid reduction to baseline after aerosol generating procedures in a mechanically ventilated environment (Shahdad et al. 2021), which is similar to the observations in Dental Clinic 2. While non-mechanically ventilated environments failed to achieve baseline levels, opening windows did reduce all particle sizes immediately (Shahdad et al. 2021). Since Dental Clinic 3 had open windows for the entire work shift, it was not possible to see the impact of opening windows following dental procedures. Additionally, particles entering through the open window could have influenced the indoor environment in this dental clinic.

Bacterial and fungal concentration levels in the present study were significantly different across the three dental clinics (Fig. S4). Furthermore, the present study utilized a DNA-based analytical technique by directly isolating DNA from actively sampled inhalable aerosol filters to determine microbial concentration levels (DNA copies/m^3^) in the breathing zone of the subject. While the CIS cassettes that were used are commonly employed for bioaerosol sampling in occupational settings (Wang et al. 2015; Mainelis 2020), little data exist on person-borne bioaerosol samplers in dental clinics (Rafiee et al. 2022). Several studies employed culture-based methods (Colony Forming Unit/m^3^) using active or passive sampling to report contamination levels (R Azari et al. 2008; Bârlean et al. 2010; Pasquarella et al. 2012; Messano et al. 2013 ; Polednik 2014; Chatoutsidou et al. 2021; Połednik 2021; Choudhary et al.2022) which may lead to a selection bias toward culturable species. Comparing microbial load with existing studies is challenging due to methodological heterogeneity. Most of these studies, however, have shown that bioaerosol contamination is higher in treatment rooms than control settings, yet there is some discrepancy (Choudhary et al. 2022; Rafiee et al. 2022). This could be due to inter-clinical variation as well as variations in choice of sampling and analysis methods. A recent systematic review and meta-analysis of aerosol formation during ultrasonic scaling confirmed that this procedure is associated with increased levels of bacterial aerosols (Gonçalves Lomardo et al. 2024 ). In agreement with these results, the current study revealed significantly higher bacterial levels in the operating rooms of both Dental Clinics 2 and 3 than in the operating room of Dental Clinic 1 (Fig. S4). In these clinics, procedures depending on pressurized air such as ultrasonic scaling, air polishing (for Dental Clinic 2) and sandblasting (for Dental Clinic 3) were more frequently carried out than in clinic 1 (Table 1). Furthermore, the results of the mixed model (Table S3) suggested that the fungal concentration and bacterial concentration differ significantly across clinics. These differences may result from distinct ventilation systems and indoor environments. Natural ventilation could lead to influx of microorganisms from outdoor air, as well as being less efficient in exchanging indoor air with fresh air than mechanical ventilation. Together with poorer indoor air quality in old buildings, these may have influenced the microbial DNA concentration levels. The comparison analysis (Fig. S4) showed that Dental Clinic 3, which was the only clinic with natural ventilation, had higher bacterial and fungal DNA concentration levels compared to Dental Clinics 1 and 2.

The three dental clinics used various products for sandblasting, air polishing, and pumice powder polishing. The electron microscopy combined with energy-dispersive X-ray analysis (Table 3) suggested that particles were released from the powders used during dental treatment procedures. Pumice stone powder used in dental prophylaxis to polish the tooth surface contains predominantly silica (SiO_2_) and alumina (Al_2_O_3_), but also other oxides such as K_2_O and Na_2_O. Individually, Si is one of the major elements of dental ceramics and Al is used in both dental ceramics (aluminum silicate) and sandblasting (aluminum oxide) procedures. The air flow powder used in Dental Clinic 2 contained nanosized amorphous silica, a nanomaterial commonly used in a variety of applications and products. Repeated intratracheal exposure resulted in local and widespread systemic effects of amorphous silica nanoparticles in mice (Li et al. 2022) and rats (Xu et al. 2025). A summary of nine occupational worker studies concluded that there is no evidence for adverse effects on the human lung (Antoniou et al. 2024), however, systemic effects have not been investigated. In addition to particles originating from dental treatment powders, elements such as Na, K, calcium (Ca), magnesium (Mg), chlorine (Cl), and phosphorus (P) can be derived from aerosolized saliva (Hatami et al. 1996; Ghadimi et al. 2013). The C-rich group may comprise a variety of organic particles, such as spores, plastic particles, and other organic particles.

The main limitation of this study is the study size. Although the sampling campaign was conducted over several days in each clinic and included several different workers, measurements were only done in one clinic relying on natural ventilation, and two clinics with mechanical ventilation systems. A validation study including several clinics within each ventilation category where the air exchange rate is additionally assessed should be done to support the current data. For instance, direct reading measurements in this study suggested that mechanical ventilation was important for efficient removal of aerosols, and data collection in a larger number of clinics could reveal whether mechanical ventilation also impacts respirable mass concentration levels. Additionally, more robust data on how the varied use of natural ventilation during warm/cold seasons contributes to influx of aerosols and bioaerosols from outdoor air, should be collected. Furthermore, microorganisms were analyzed with pan-specific markers for bacteria and fungi. Adding viral sampling and performing deeper analyses of specific species would provide information on pathogenicity and aid in the interpretation of the data considering possible environmental (outdoor/indoor air, dental unit water) and human (oral/skin) sources. Understanding the microbial landscape in a dental clinic and how it is influenced by factors such as ventilation and seasonal variations could support targeted infection control strategies and overall improve health and safety measures for both patients and dental personnel.

## Conclusion

All collected respirable samples in the three dental clinics were well below the Norwegian occupational exposure limit for respirable dust currently set to 5 mg/m^3^ (Arbeidstilsynet 2024). Despite low respirable particle mass concentrations detected in the breathing zone of dental health care workers, this study revealed a high number concentration of ultrafine particles, stressing the potential risk of occupational respiratory and systemic diseases. Particularly, particle numbers were episodically elevated in clinics dominated by prophylactic and restorative procedures, and their shorter suspension time was concurrent with the presence of a mechanical ventilation system. Additionally, levels of inhalable bacterial and fungal DNA seemed to be associated with clinical characteristics. Taken together, this highlights the need for systematic studies on the use of mechanical versus natural ventilation in a larger number of dental clinics and their effect on aerosol exposure. Furthermore, extended studies are needed to pinpoint exact levels of particle exposure and to map the potential health outcomes of dental health care personnel exposed to respirable particles and bioaerosol in dental clinics.

## Supplementary Material

wxaf073_Supplementary_Data

## Data Availability

The data supporting the results of the study are accessible upon reasonable request to the corresponding author.
