# Supplementary material for: Characterization of occupational exposure to airborne particles and bioaerosols in dental clinics
Source: Ann Work Expo Health. 2025 Nov 4;70(1):wxaf073. doi: 10.1093/annweh/wxaf073 (PMC12821372; doi:10.1093/annweh/wxaf073)
Supplement: wxaf073_Supplementary_Data [file wxaf073_supplementary_data.pdf]

Supplementary material for:

Characterization of occupational exposure to airborne particles and bioaerosols in dental clinics

Rubiyat E. Islam[1,2], Lina Wik[1], Vibeke E. Ansteinsson[2,3], Pål Graff[1], Shan Zienolddiny-Narui[1], Torunn K. Ervik[1]

[1] National Institute of Occupational Health (STAMI), Oslo, Norway

[2] Department of Public Health Science, Institute of Health and Society, Faculty of Medicine, University of Oslo (UiO)

[3] Oral Health Centre of Expertise in Eastern Norway (OHCE), Oslo, Norway.

Corresponding author: lina.wik@stami.no

Supplementary Table S1: Overview of sampling days and the number of samples collected each day. Each worker was always carrying the samplers for respirable dust and bioaerosol in parallel, hence the numbers are valid for both particle and bioaerosol samples.

**Clinic 1**	**1**	**2**	**3**	**4**	**5**	**6**	**7**	**8**	**9**	**Total samples**
Dentist	1	1	1	1	1	1	1	1	1	9
Dental health secretary	1	1	1		1	1	1	1	1	8
Dental hygienist				1						1
Sum										18
**Clinic 2**										
Dentist	3	3	3	3						12
Dental health secretary	1	1		2						4
Sum										16
**Clinic 3**										
Dentist	1	2	2	1						6
Dental health secretary	1		1	1						3
Sum										9

Supplementary Table S2: Description of the performed dental procedures and their potential for particle generation.

**Mechanisms of particle generation during various dental procedures. Procedure**	**Performed procedures in dental clinics**	**Description of the procedures**	**Particle generation**
**Ultrasonic scaling**	All three dental clinics participated in the present study used ultrasonic scalers for the scaling treatment procedure.	Dental professionals widely use ultrasonic scalers as an efficient instrument to remove plaque, calculus, and stain from teeth. This scaler utilizes high frequency sound to generate rapid vibration at the tip, effectively breaking apart plaque and calculus from the surface of teeth and along the gum line. The scaler produces fine spray of water to prevent overheating of the tip, irrigates the treatment area by washing away debris, and enhances the cleaning process by keeping the area moist (X., 2024).	Using Ultrasonic scaler during dental treatment can generate particles along with droplets from splashing, which may become aerosolized as droplet nuclei and released into the air of the clinic. Droplets larger than 100 µm settle on the torso and head of patient, as well as on the floor and dental chair, contaminating the dental operating area at rates as high as 78.5%(Zhang et al., 2023). During certain surgical procedures, dentists work closer to a patient's mouth, which can block dispersion, causing droplets to focus on the dentist's mask and gown(Veena et al., 2015). When the droplets evaporate from surfaces, they transform into droplets nuclei (< 5 µm) that become airborne and carries microbial agents, such as bacteria, fungi or viruses. These nuclei have the potential to remain airborne longer and spread diseases over greater distances (Zhang et al., 2023).
**Sandblasting**	Among the three dental clinics, Dental Clinic 3 conducted the sandblasting procedure using aluminum oxide	Sandblasting in dentistry refers to the use of a high-pressure stream of abrasive materials, such as aluminum oxide to clean or prepare tooth surfaces for	The operating principles of sandblasting works by high-speed alumina particles to create abrasive energy when they hit the surface of the tooth. Although the particles

	particles (Rønvig Dental Mfg. AS, Daugaard, Denmark).	restorative procedures (Baumgartner et al., 2017). The impact of the abrasive particles can create fine dust and aerosolize small particles from the treated areas, which may include s contaminated aluminum oxide particles (Roeters, 2000).	are very small in size, they exit the nozzle at a high speed. When the particles strike the tooth surfaces with high energy, they micro-abrade the tooth as well as dislodge the small particles of decay and other tooth materials. This process generates fine particles that can easily become airborne (Freedman, 2012).
**Air polishing**	Dental Clinic 2 mostly used Sodium bicarbonate-based ($NaHCO_3$) AirFlow powder (EMS, Nyon, Switzerland) for air polishing treatment procedure. Pumice powder polishing on tooth surface was performed both in Dental Clinic 1 and 3.	An alternative treatment method for eliminating plaque, extrinsic stains and deposits located above the gum line of teeth is known as air polishing. This method employs a lightweight handpiece similar to an ultrasonic scaler to create a mixture of pressurized air, abrasive powder, and water for the removal of plaque biofilm and stain. Sodium bicarbonate-based powder ($NaHCO_3$), glycine, calcium sodium phosphosilicate, calcium carbonate and aluminum trihydroxide are commonly used powder materials in air polishing treatment (Graumann et al., 2013).	The handpiece of the air powder polisher can be connected directly to the air/water connector of the dental unit, used as an independent unit, or integrated alongside ultrasonic sealers. The stand-alone air polishing device utilizes a swirling mechanism to combine air and powder, whereas the handpiece version produces the air and powder mixture through a carburetor method combined with swirling. The quantity of powder released through aerosolization varies with different settings on the air polishing device, which is determined by how the pressurized air is directed through the powder chamber. This variation leads to notable differences in powder emission rates among different air polishing devices models at various powder settings (Chowdhary, 2015).
**Composite filling restoration and polishing**	Composite filling restoration followed by polishing and reshaping of the restoration were performed in all the three dental clinics.	Resin-based dental composite has become a crucial part in restorative to replace and restore the decayed portion of tooth structures (Lien & Vandewalle, 2010). A composite restoration is composed of inorganic filler particles, an organic resin polymer matrix as well as silane coupling agent that helps to bind	Generation of aerosolized particles during dental restoration procedures occurs through several processes. Dentists use high speed coarse and fine drilling instruments. This may lead increase heat generation and the release of particles due to the thermal decomposition of the composite polymer matrix caused by the

		the filler particles to methacrylate resin matrix during polymerization process (Lien & Vandewalle, 2010). In the course of standard dental procedures, such as finishing and polishing composite filing restoration or replacement of old composite restoration or debonding brackets in orthodontics, dental health personnels are exposed and inhale aerosolized composite dust in a daily working environment (Iliadi et al., 2020)	frictional heat (Bradna et al., 2017; Camassa et al., 2021). In addition, Van Landuyt et al. showed that nanoparticles can originate from single nanofiller particles and release during polishing of dental composite (Van Landuyt et al., 2014; Van Landuyt et al., 2012). Although the composite restoration causes particle formation in dental clinics, the frequency of the procedure plays a significant role in determining the quantity of particles produced. In the present study, the representative dental clinics performed fewer composite restorations compared to ultrasonic scaling, which may lead to lower particle concentration resulting from the composite restoration.
**Cement crown preparation**	Mostly Dental Clinic 2 and 3 performed cement crown preparation.	In dentistry, Crown restoration is done to rehabilitate and restore teeth to maintain physiological health (McCracken et al., 2016). The crown placement is indicated in endodontic procedures, tooth cracks and fractures, large restorative needs, aesthetic deficiencies or in carious lesions (Bader et al., 2004; Bader et al., 1996; McCracken et al., 2016).	During the crown preparation process, aerosolization of particles can occur. During the placement of the prepared crown onto the tooth using dental cement can generate aerosols in the dental environment (Iliadi et al., 2020). However, the aerosol formation during cementing crown is minimal than composite restoration and other dental procedures which use high speed instruments and the mechanical manipulation of materials, resulting in greater potential for airborne particles.

References for Supplementary Table S2:

Bader, J. D., Shugars, D. A., & Martin, J. A. (2004). Risk indicators for posterior tooth fracture. J Am Dent Assoc, 135(7), 883-892. https://doi.org/10.14219/jada.archive.2004.0334.

Bader, J. D., Shugars, D. A., & Roberson, T. M. (1996). Using crowns to prevent tooth fracture. Community Dent Oral Epidemiol, 24(1), 47-51. https://doi.org/10.1111/j.1600-0528.1996.tb00812.x.

Baumgartner, S., Koletsi, D., Verna, C., & Eliades, T. (2017). The Effect of Enamel Sandblasting on Enhancing Bond Strength of Orthodontic Brackets: A Systematic Review and Meta-analysis. J Adhes Dent, 19(6), 463-473. https://doi.org/10.3290/j.jad.a39279.

Bradna, P., Ondrackova, L., Zdimal, V., Navratil, T., & Pelclova, D. (2017). Detection of nanoparticles released at finishing of dental composite materials. Monatshefte für Chemie - Chemical Monthly, 148(3), 531-537. https://doi.org/10.1007/s00706-016-1912-6.

Camassa, L. M. A., Ervik, T. K., Zegeye, F. D., Mdala, I., Valen, H., Ansteinsson, V., & Zienolddiny, S. (2021). Characterization and toxicity evaluation of air-borne particles released by grinding from two dental resin composites in vitro. Dental Materials, 37(7), 1121-1133. https://doi.org/https://doi.org/10.1016/j.dental.2021.03.011.

Chowdhary, Z. (2015). Air Polishing: An Update. International Journal of Maxillofacial Research, 1, 34-46.

Freedman, G. (2012). Chapter 26 - Technology and Esthetics. In Contemporary Esthetic Dentistry (pp. 582-642). Mosby. https://doi.org/https://doi.org/10.1016/B978-0-323-06895-6.00026-8.

Graumann, S. J., Sensat, M. L., & Stoltenberg, J. L. (2013). Air polishing: a review of current literature. *J Dent Hyg*, *87*(4), 173-180.

Harrel, S. K., & Molinari, J. (2004). Aerosols and splatter in dentistry: a brief review of the literature and infection control implications. J Am Dent Assoc, 135(4), 429-437. https://doi.org/10.14219/jada.archive.2004.0207.

Iliadi, A., Koletsi, D., Eliades, T., & Eliades, G. (2020). Particulate Production and Composite Dust during Routine Dental Procedures. A Systematic Review with Meta-Analyses. Materials (Basel), 13(11). https://doi.org/10.3390/ma13112513.

Lien, W., & Vandewalle, K. S. (2010). Physical properties of a new silorane-based restorative system. Dental Materials, 26(4), 337-344. https://doi.org/https://doi.org/10.1016/j.dental.2009.12.004.

McCracken, M. S., Louis, D. R., Litaker, M. S., Minyé, H. M., Mungia, R., Gordan, V. V., Marshall, D. G., & Gilbert, G. H. (2016). Treatment recommendations for single-unit crowns: Findings from The National Dental Practice-Based Research Network. J Am Dent Assoc, 147(11), 882-890. https://doi.org/10.1016/j.adaj.2016.06.012.

Roeters, J. J. (2000). A simple method to protect patient and environment when using sandblasting for intraoral repair. J Adhes Dent, 2(3), 235-238.

Van Landuyt, K. L., Hellack, B., Van Meerbeek, B., Peumans, M., Hoet, P., Wiemann, M., Kuhlbusch, T. A., & Asbach, C. (2014). Nanoparticle release from dental composites. Acta Biomater, 10(1), 365-374. https://doi.org/10.1016/j.actbio.2013.09.044.

Van Landuyt, K. L., Yoshihara, K., Geebelen, B., Peumans, M., Godderis, L., Hoet, P., & Van Meerbeek, B. (2012). Should we be concerned about composite (nano-)dust? Dental Materials, 28(11), 1162-1170. https://doi.org/https://doi.org/10.1016/j.dental.2012.08.011.

Veena, H. R., Mahantesha, S., Joseph, P. A., Patil, S. R., & Patil, S. H. (2015). Dissemination of aerosol and splatter during ultrasonic scaling: a pilot study. J Infect Public Health, 8(3), 260-265. https://doi.org/10.1016/j.jiph.2014.11.004.

X., Xiezhangjiang. (2024). The difference between ultrasonic scaler and air scaler? Retrieved 28th April from https://izidental.com/blogs/news/the-difference-between-ultrasonic-scaler-and-air-scaler.

Zhang, F., Zhao, J., Yang, W., Yu, X., He, J., Shu, H., & Zhu, X. (2023). Studying the spatial flow characteristics of droplets induced by ultrasonic tooth-cleaning machines. Physics of Fluids, 35. https://doi.org/10.1063/5.0171481.

Supplementary Table S3: The association between respirable particle mass concentration, total bacterial and total fungal DNA concentration and worker classification were analyzed by linear mixed models (LMMs). The response variables were particle concentration, bacterial DNA, and fungal DNA concentration, with profession specified as a fixed effect and dental clinic as a random effect.

	Particle concentration (n=39)			*Bacterial concentration (n=43)*			*Fungal concentration (n=43)*		
	Estimate (**β**)	Standard error	P-value	Estimate (**β**)	Standard error	P-value	Estimate (**β**)	Standard error	P-value
Intercept	0.024	0.0047	0.0015	3.6	0.25	0.0028	3.0	0.36	0.012
Fixed effect									
Profession	-0.001	0.0057	0.84	0.021	0.11	0.85	0.11	0.090	0.25
Random effects									
Variance (Intercept for Clinic)	7.5e-06			0.16			0.36		
Standard Deviation (Clinic Intercept)	0.0027			0.40			0.60		
Residual Variance	3.0e-04			0.11			0.081		
Residual Standard Deviation	0.017			0.34			0.29		

Supplementary Figure S1

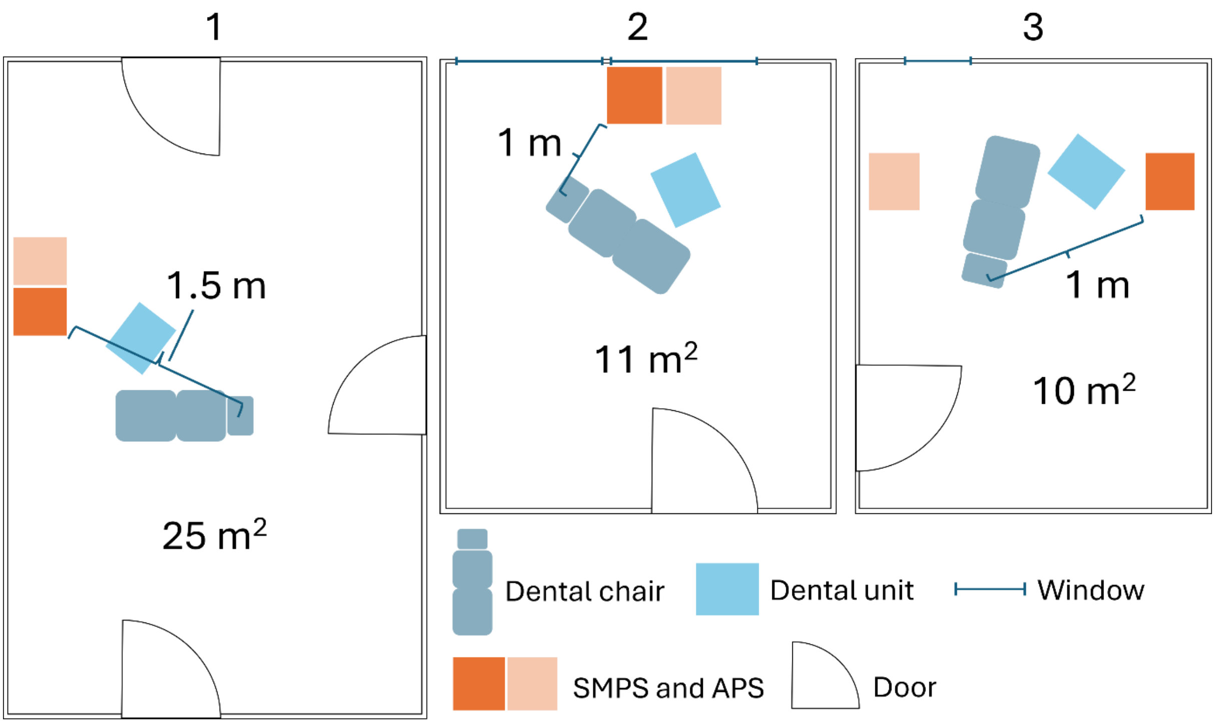

Figure S1. Floor plan of treatment rooms in Dental Clinic 1, 2 and 3, respectively, showing placement of relevant items and distances from the SMPS and APS to the position of the patient head. Windows in Dental Clinic 2 were never opened. Drawings may not be in exact scale.

Supplementary Figure S2

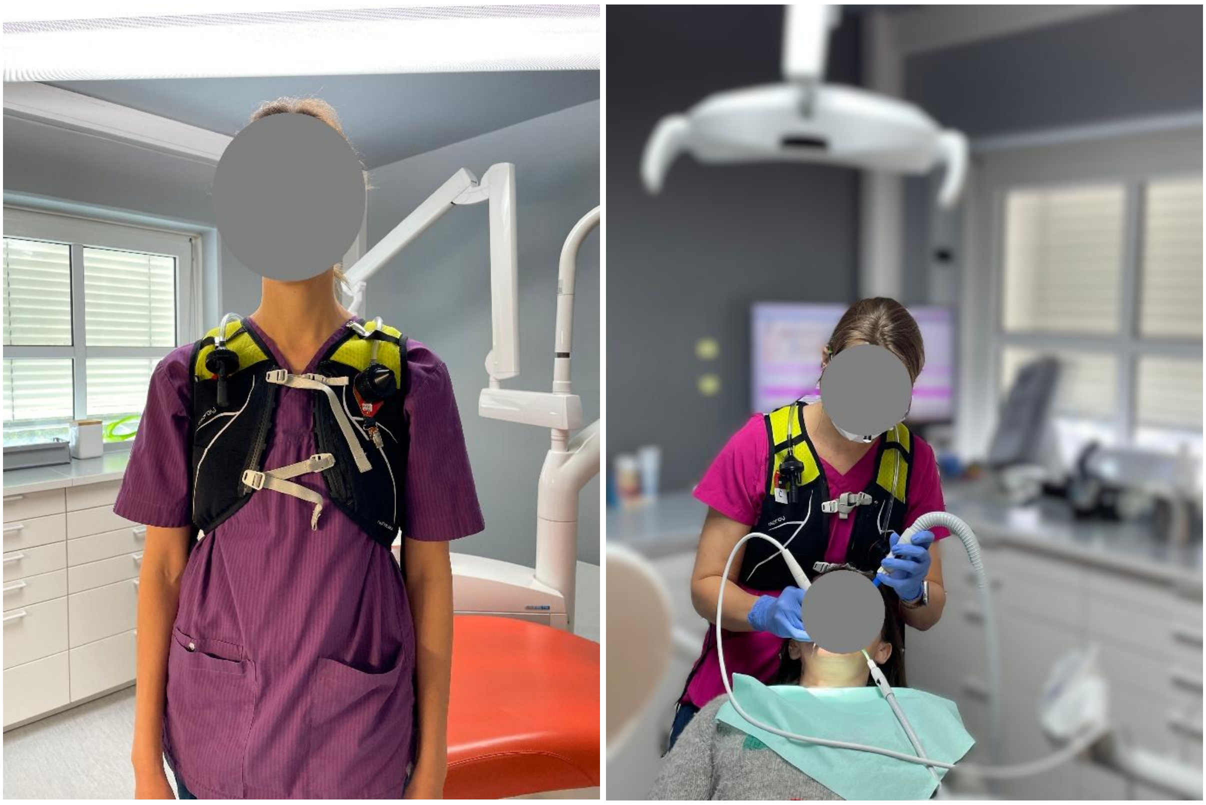

Figure S2: Dental workers wearing person-borne sampling equipment with pumps carried in a backpack before (left) and during (right) dental procedures. The respirable cyclone and conical inhalable sampler are attached within the breathing zone.

Supplementary Figure S3

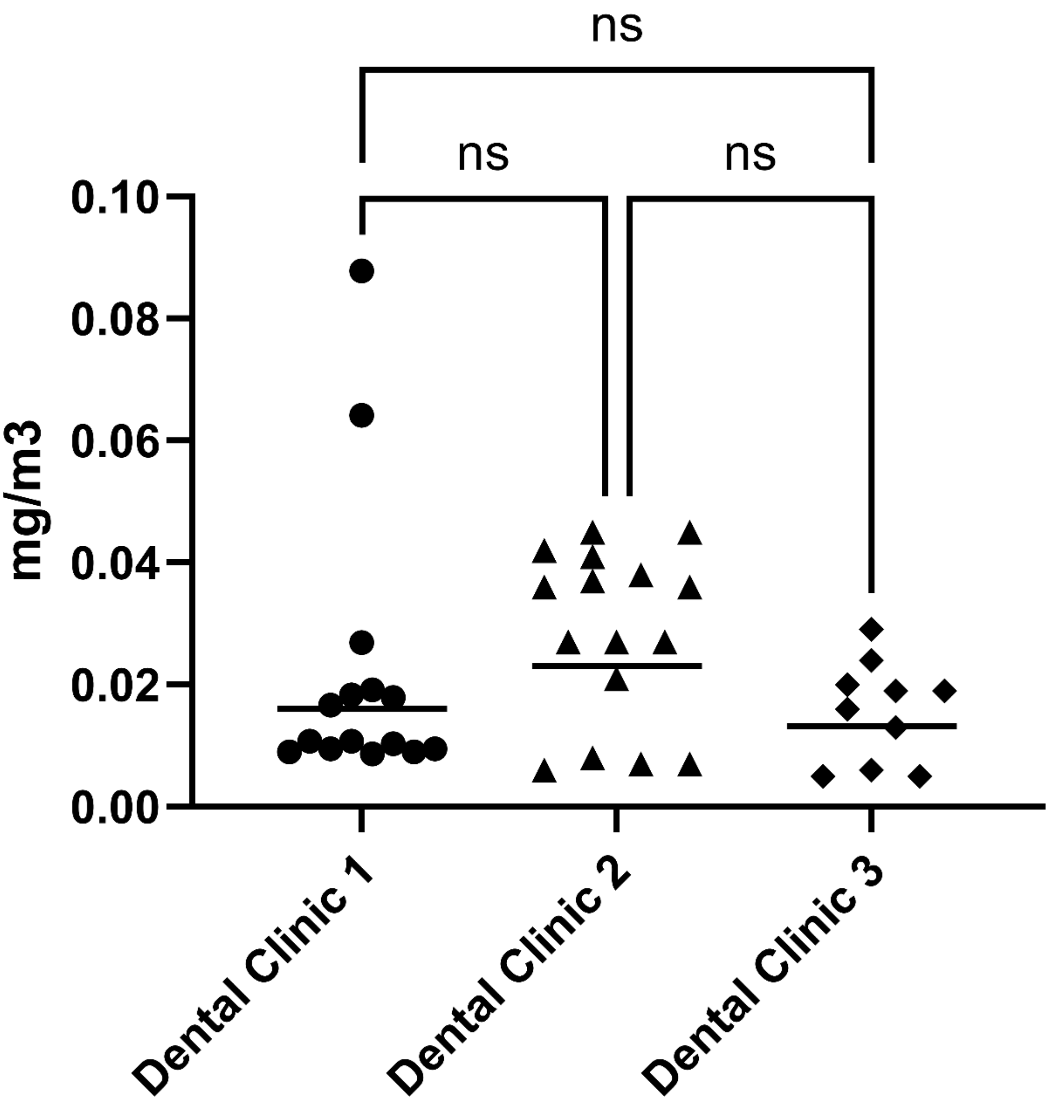

Figure S3. Comparison of GM personal respirable particle mass concentration (mg/m^3) among three dental clinics. The horizontal bars represent the GM of each group. Particle mass concentration varied between Dental Clinic 1 and 2 as well as between Dental Clinic 2 and 3, however, these differences were not statistically significant (P = 0.5698, P = 0.1775, respectively). Analysis (ANOVA followed by Tukey's multiple comparison test) was performed on log-transformed data.

Supplementary Figure S4

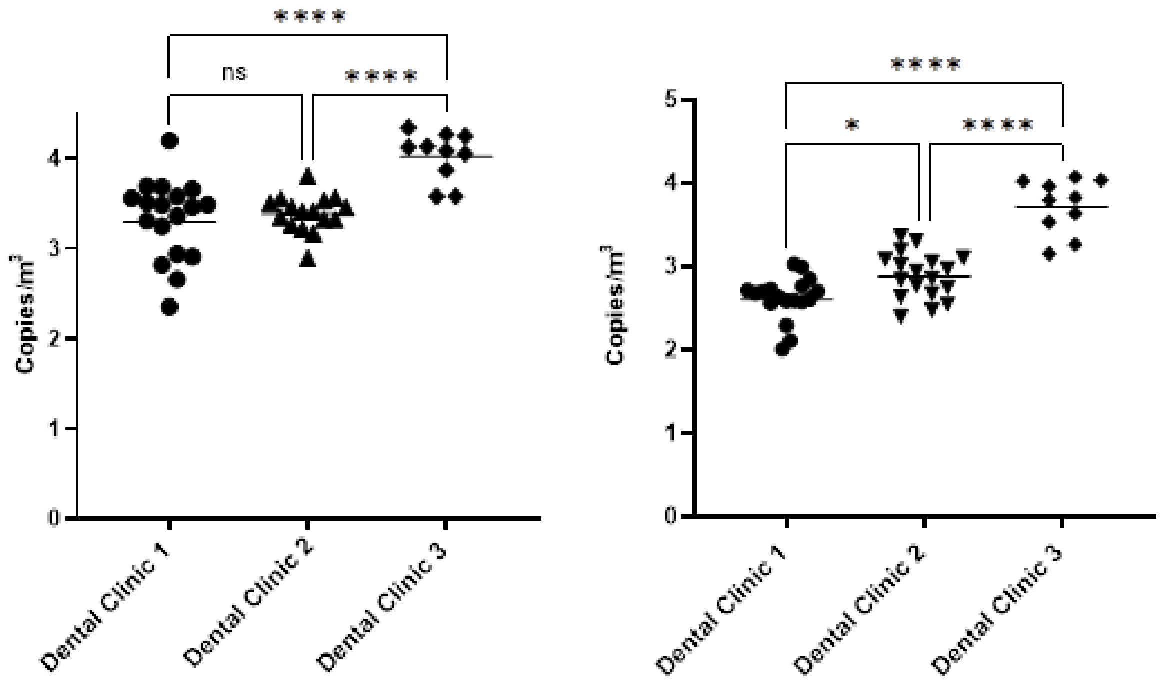

A B

Figure S4. Comparison of A) GM of total bacterial DNA concentration (copies/m^3) among three dental clinics and B) GM of total fungal DNA concentration (copies/m^3) among three dental clinics collected by personal inhalable samplers (log transformed data, ANOVA followed by Tukey's multiple comparison test). The horizontal bars represent the GM of each group. The geometric mean exposure levels of total bacterial DNA and total fungal DNA concentration differed significantly among dental clinics (P < 0.0001). Statistical differences in bacterial DNA concentration were found between Dental Clinic 1 and 3 (P < 0.0001) and between Dental Clinic 2 and 3 (P < 0.0001), but not between Dental Clinic 1 and 2. Statistical differences in fungal DNA concentration were found between Dental Clinic 1 and 3 (P < 0.0001), Dental Clinic 2 and 3 (P < 0.0001), and Dental Clinic 1 and 2 (P < 0.05). Significance levels * < 0.05, ** < 0.01, *** < 0.001, **** < 0.0001.

Supplementary Figure S5

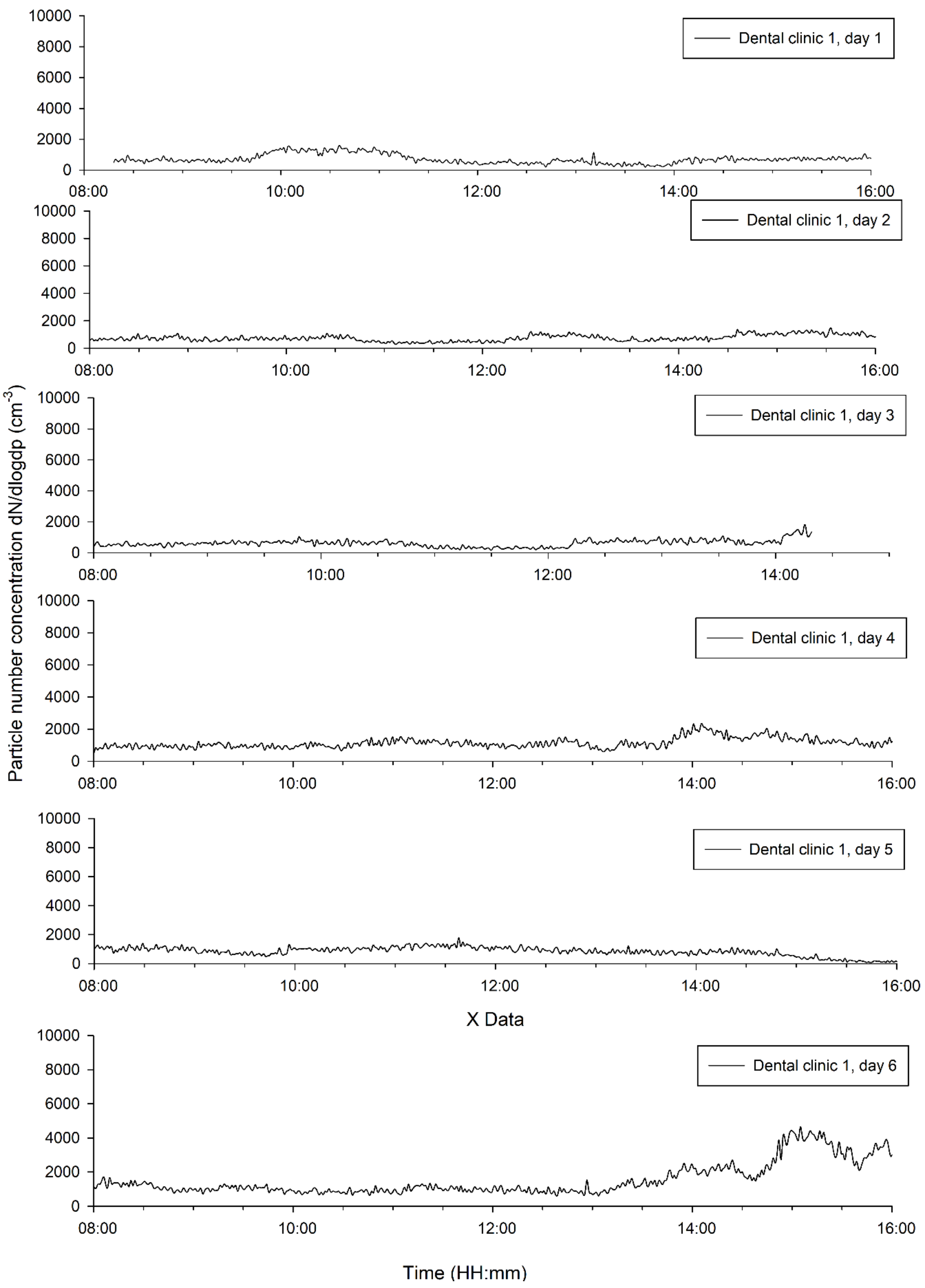

Figure S5: SMPS-time series of the total number concentration for particles in the size range of 16.8-593 nm for the first six sampling days in Dental clinic 1.

Supplementary Figure S6

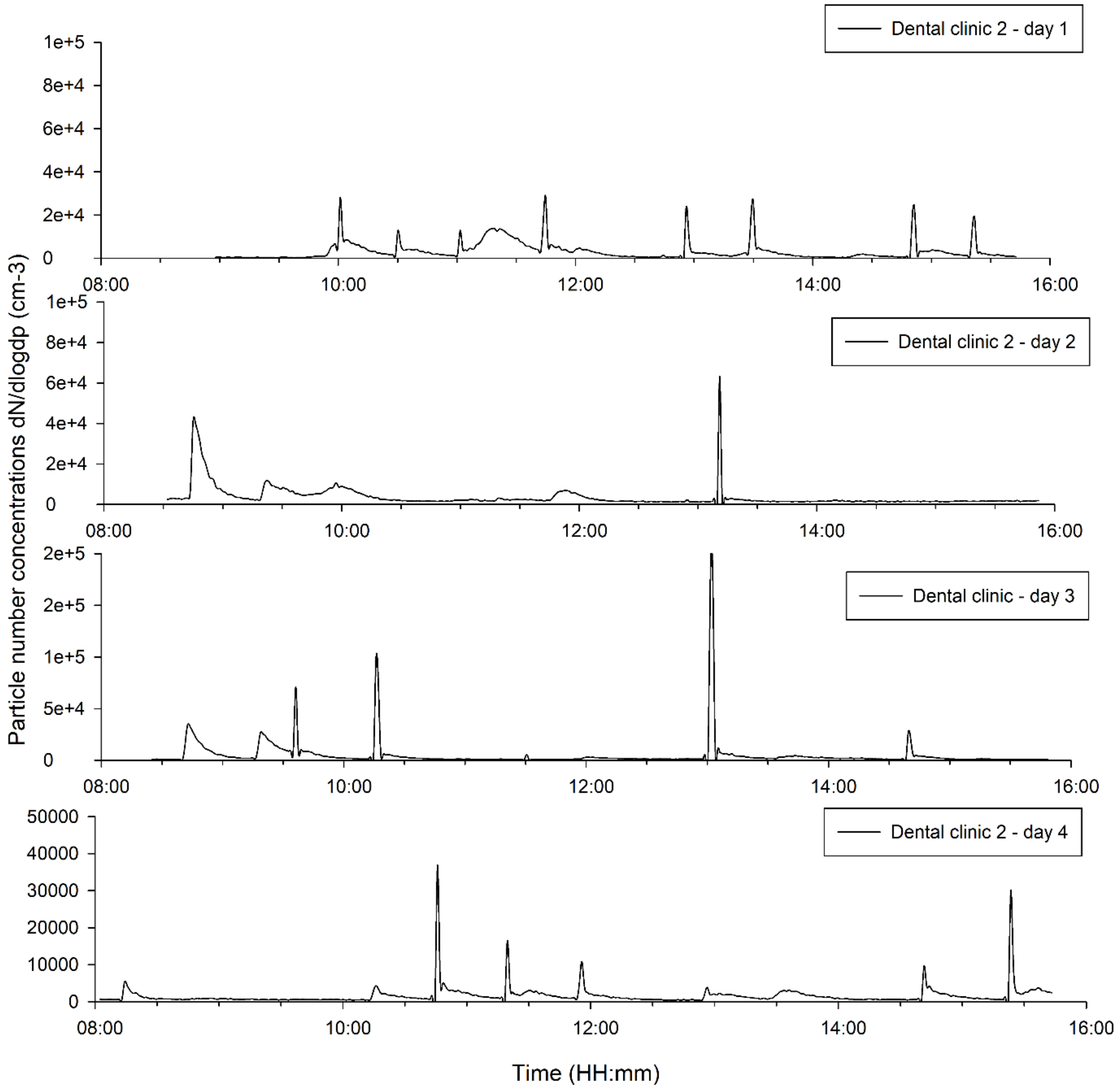

Figure S6: SMPS-time series of the total number concentration for particles in the size range of 16.8-593 nm for all sampling days Dental clinic 2.

Supplementary Figure S7

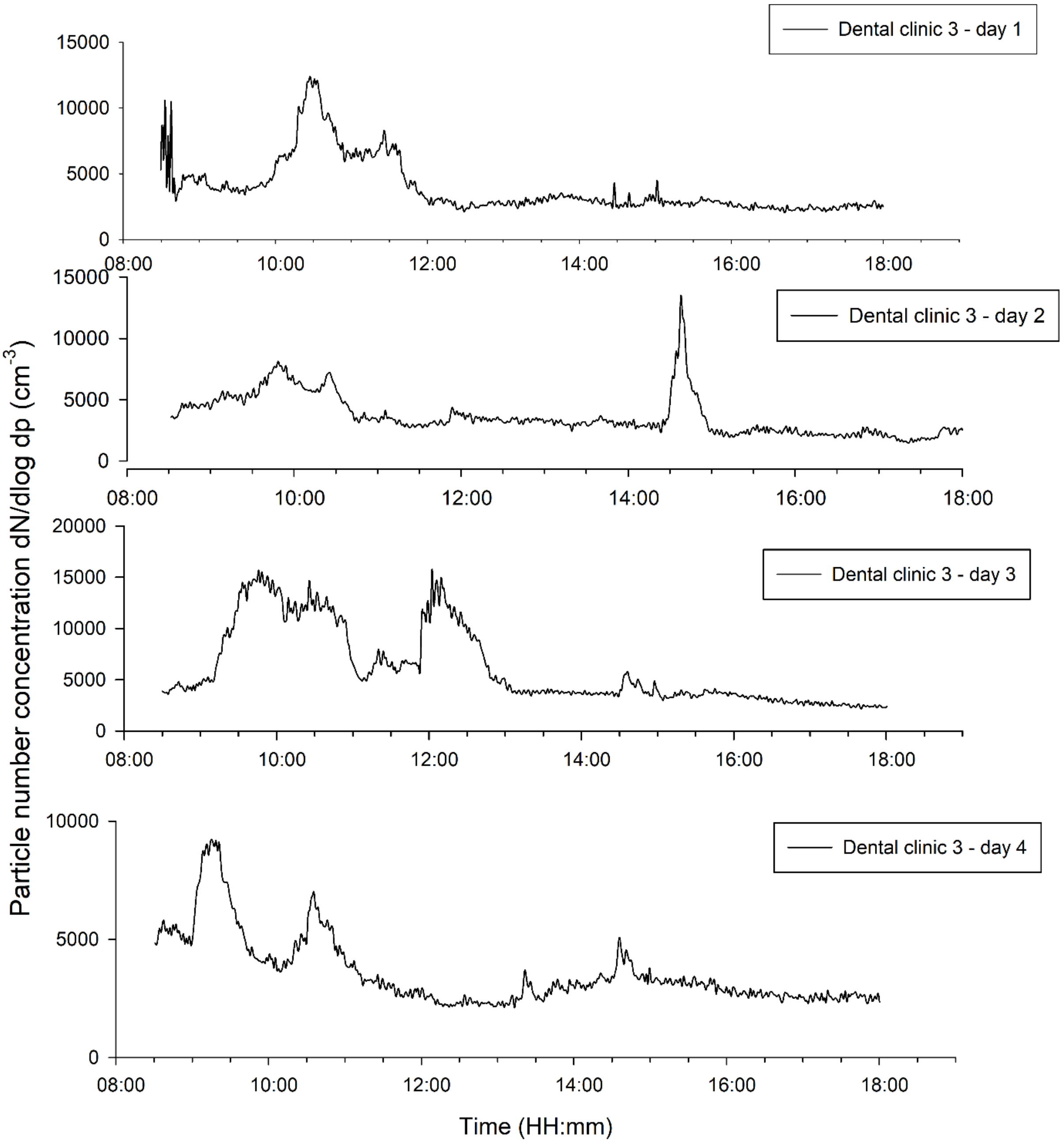

Figure S7: SMPS-time series of the total number concentration for particles in the size range of 16.8-593 nm for all sampling days Dental clinic 3.

Supplementary figure S8

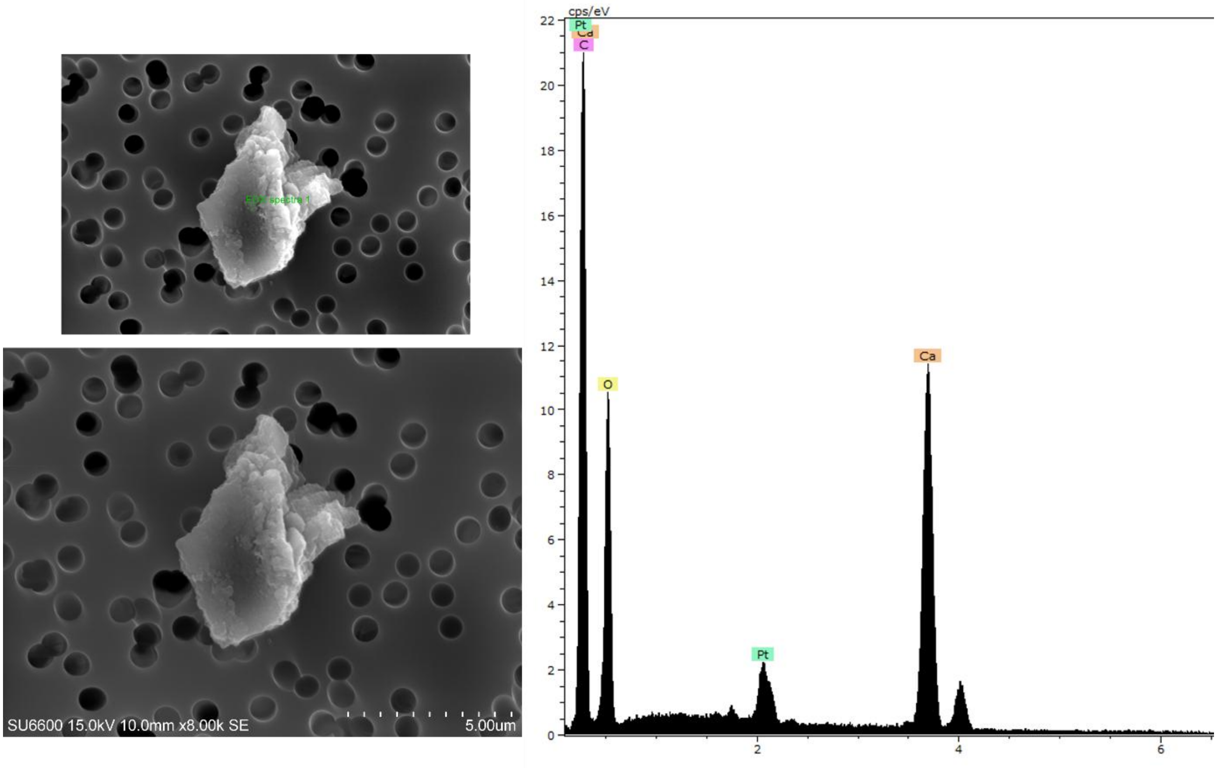

A. Ca-rich particle

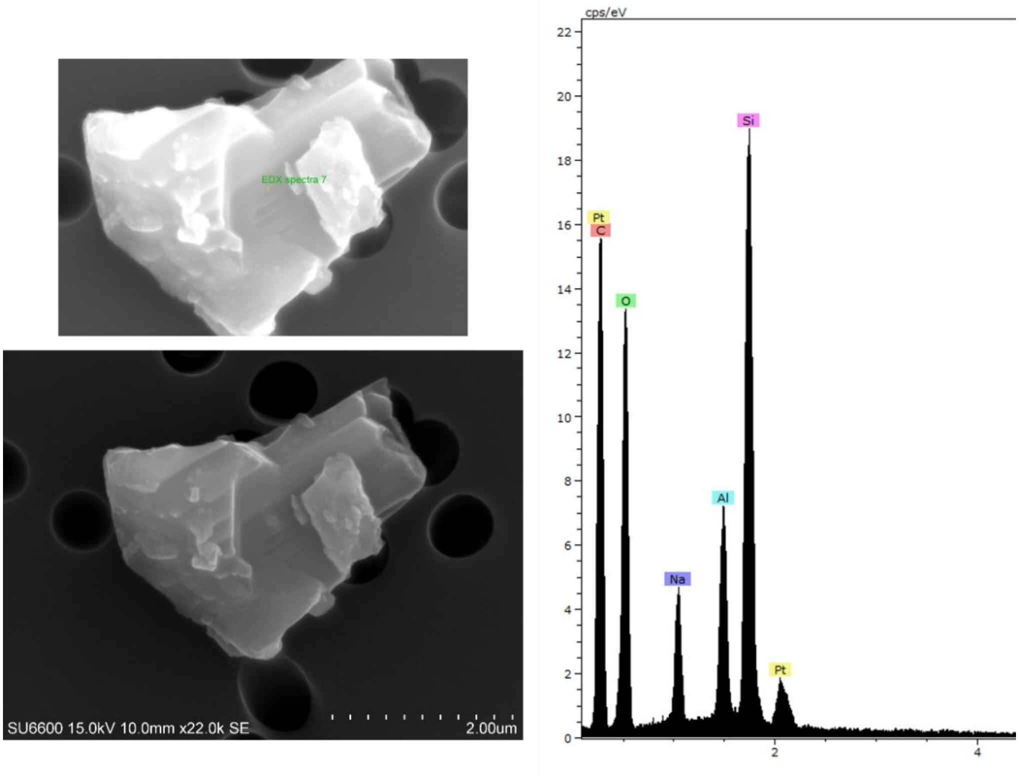

B. Airflow powder

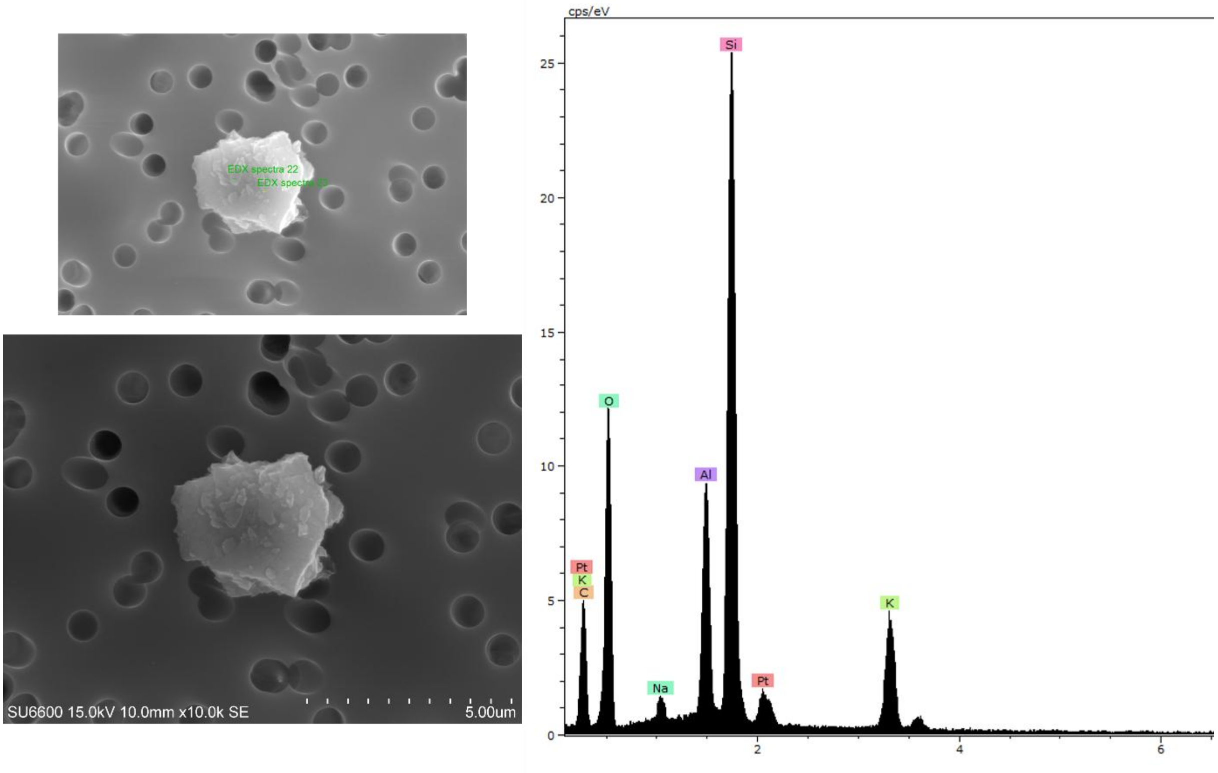

C. Pumice particle

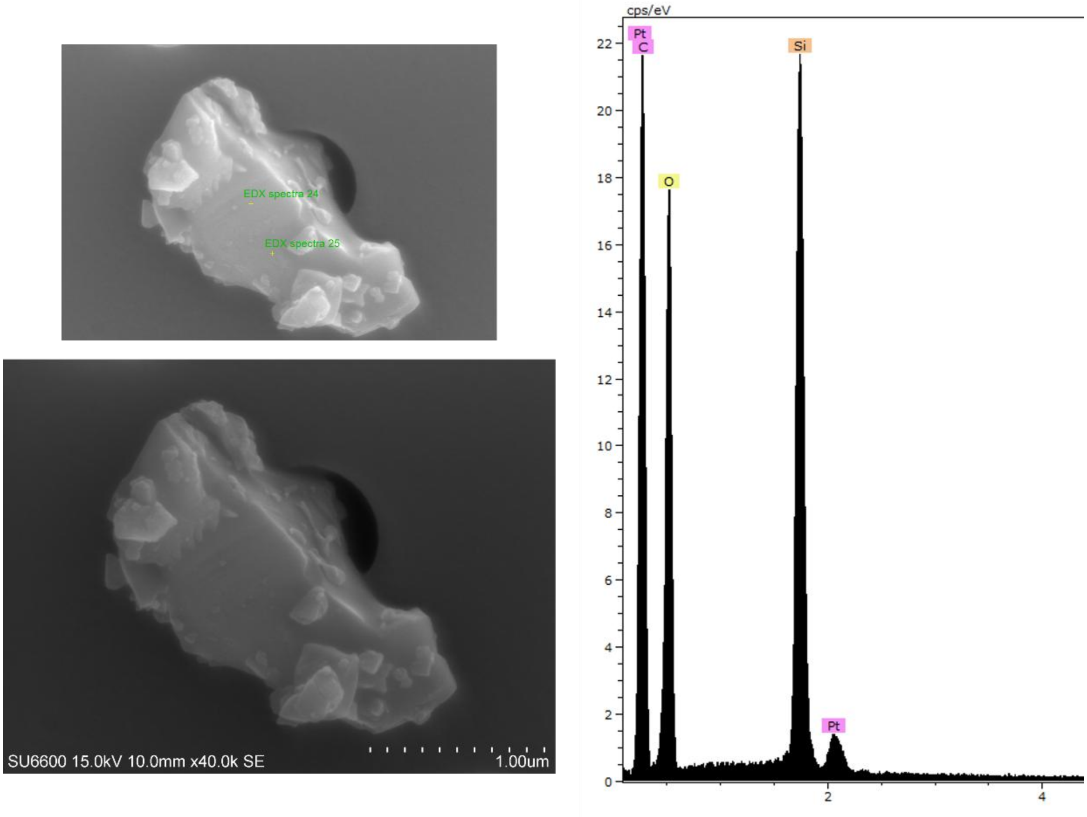

D. Si-rich particle

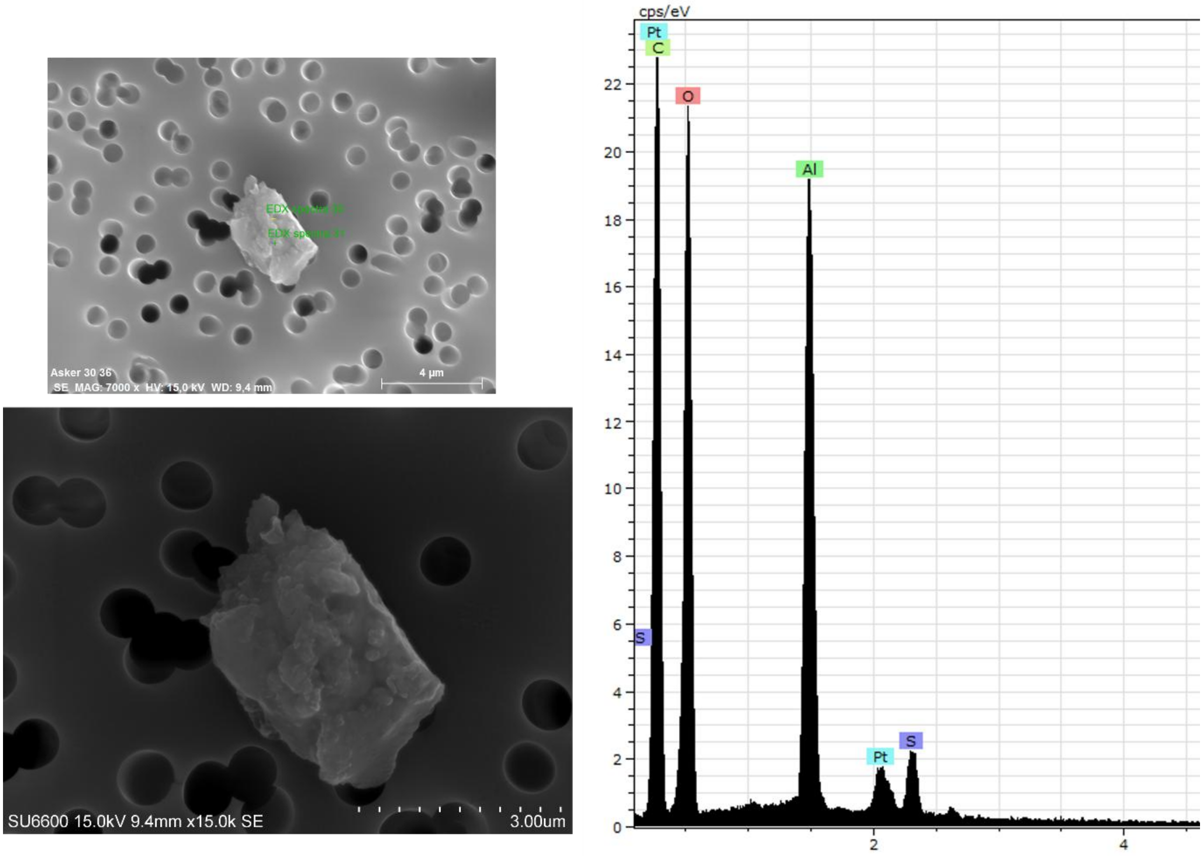

E. Al-rich particle

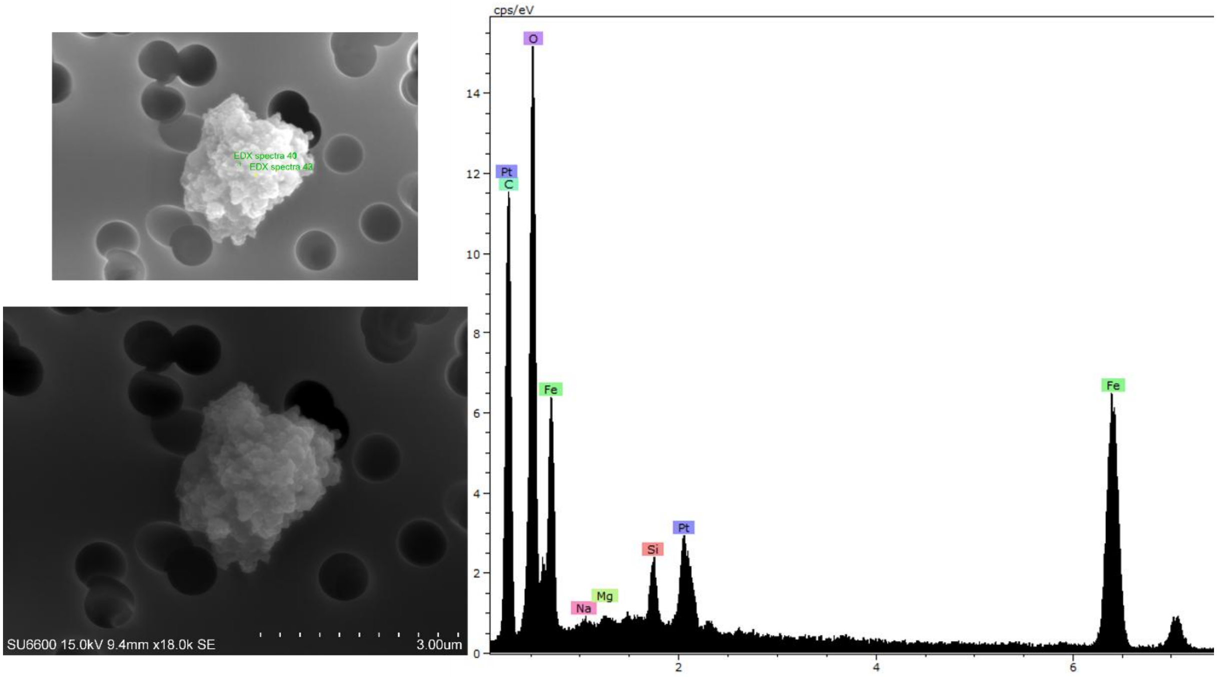

F. Fe-rich particle

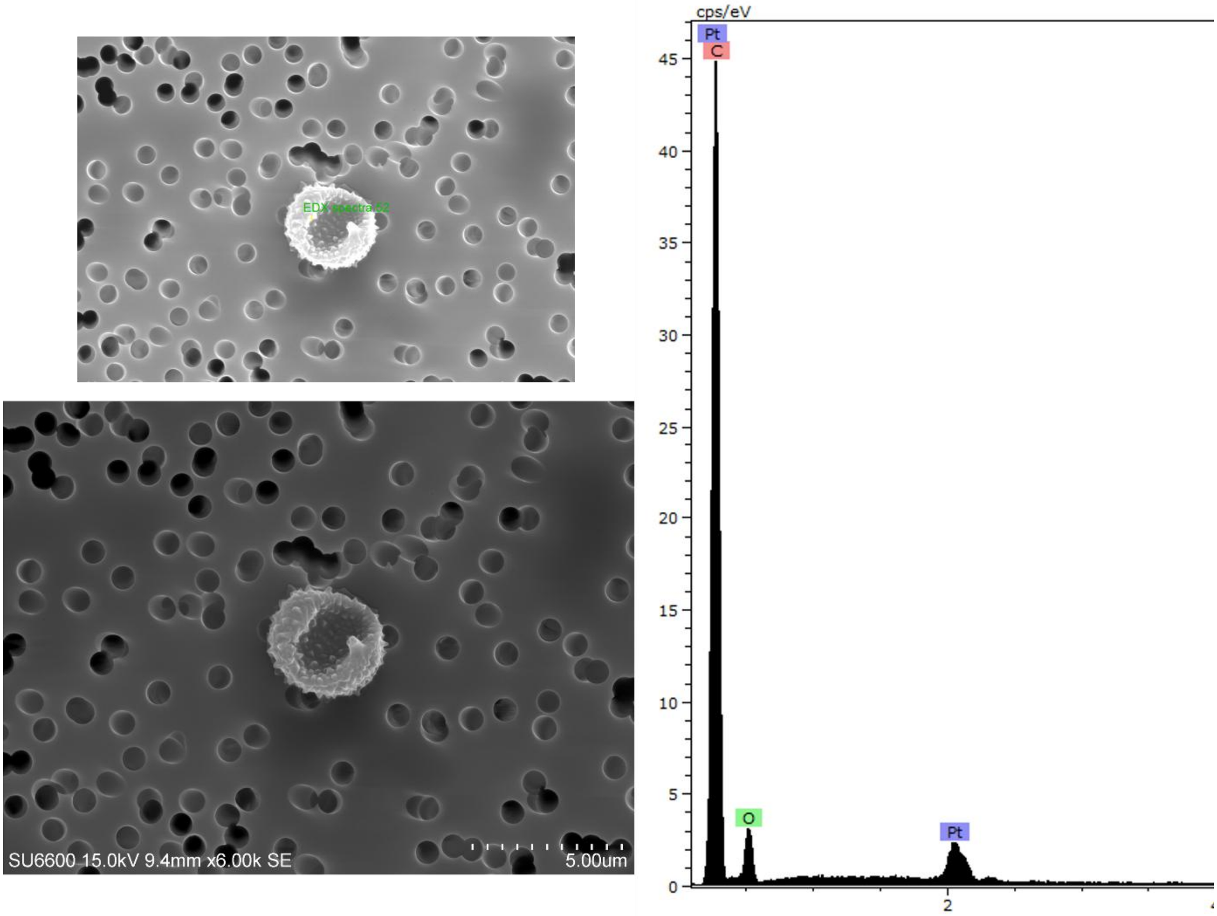

G. Particle collected at Dental Clinic 3 with high C-content.

Figure S8. SEM images and associated EDX spectra of airborne particles collected at the dental clinics with personal respirable samplers. Pt comes from the coating **A.** Particle collected at Dental Clinic 2 with high Ca-content. **B.** Particle collected at Dental Clinic 2 with composition similar to airflow powder. **C.** Particle collected at Dental Clinic 3 with elemental composition similar to Pumice stone powder. **D.** Particle collected at Dental Clinic 2 with high Si content. **E.** Particle collected at Dental Clinic 3 with high Al content. **F.** Particle collected at Dental Clinic 3 with high Fe content. **G.** Particle collected at Dental Clinic 3 with high C-content. The morphology of this particle points towards a spore.

ALT text Supplementary Tables and Figures

Table S1. This table provides a breakdown of the samples collected at each day and clinic and by each professional category. In total 43 samples were collected for respirable dust and bioaerosol, respectively.

Table S2. This table provides a description of the procedures that were performed during the sampling campaign, and in which clinic they were performed at. Additionally, the table provides the particle generation capacity of each procedure listed.

Table S3. This table provides the results of the linear mixed models. This analysis was performed in order to assess whether the outcome (respirable particle, bacterial or fungal DNA concentration) was influenced by worker classification (dentist or dental health secretary), with dental clinic as random effect.

Figure S1. A figure of the floor plan of treatment rooms in Dental Clinic 1, 2 and 3, respectively, showing placement of the SMPS and APS, the distances from the SMPS and APS to the position of the patient head as well as placement of windows and doors. The size of the room is indicated in square meters.

Figure S2: Pictures of a dental worker while wearing personal sampling equipment for respirable dust and bioaerosol. The picture shows how the samplers are attached to the worker before and during work.

Figure S3. Scatter plot showing a comparison of the personal respirable particle mass concentration (mg/m^3) between the three dental clinics. Geometric mean is represented by horizontal lines. Particle mass concentration was not statistically different between Dental Clinics.

Figure S4. Scatter plot showing a comparison of bacterial (A) and fungal (B) DNA concentration in copies/m^3 between the three dental clinics. Samples were collected by personal inhalable samplers. The horizontal bars represent the geometric mean of each group. The statistical analyses found significant differences in bacterial DNA concentrations between Dental Clinic 1 and 3, and Dental Clinic 2 and 3. Statistical differences were also found for fungal DNA concentrations between Dental Clinic 1 and 3, and Dental Clinic 2 and 3. Significance levels (* < 0.05, ** < 0.01, *** < 0.001, **** < 0.0001) are indicated by asterisks.

Figure S5: Plot showing SMPS-time series of the total number concentration for particles in the size range of 16.8-593 nm for the first six sampling days in Dental clinic 1.

Figure S6: Plot showing SMPS-time series of the total number concentration for particles in the size range of 16.8-593 nm for all sampling days Dental clinic 2.

Figure S7: Plot showing SMPS-time series of the total number concentration for particles in the size range of 16.8-593 nm for all sampling days Dental clinic 3.

Figure S8. Pictures numbered A-G of different particles collected during sampling with personal respirable samplers. Images are taken with scanning electron microscopy (SEM) and associated energy dispersive x-ray (EDX) spectra of the particles are shown next to each

corresponding picture. The EDX spectra indicate the chemical composition of the particles. All particles shown are of inorganic source except the last, which is Carbon rich and morphologically resembles a spore.
